# Redox-Active Water-Soluble Low-Weight and Polymer-Based Anolytes Containing Tetrazine Groups: Synthesis and Electrochemical Characterization

**DOI:** 10.3390/polym17010060

**Published:** 2024-12-29

**Authors:** Elena Yu. Kozhunova, Vyacheslav V. Sentyurin, Alina I. Inozemtseva, Anatoly D. Nikolenko, Alexei R. Khokhlov, Tatiana V. Magdesieva

**Affiliations:** 1 Physics Department, Lomonosov Moscow State University, Moscow 119991, Russia; sentyurinvv@gmail.com (V.V.S.); a.i.inozemtseva@yandex.ru (A.I.I.); nikolenko2001@gmail.com (A.D.N.);; 2 Chemistry Department, Lomonosov Moscow State University, Moscow 119991, Russia; 3 N.N. Semenov Federal Research Center for Chemical Physics, Moscow 119991, Russia

**Keywords:** redox-active materials, polymers, microgels, tetrazines, redox flow batteries

## Abstract

Polymer-based aqueous redox flow batteries (RFBs) are attracting increasing attention as a promising next-generation energy storage technology due to their potential for low cost and environmental friendliness. The search for new redox-active organic compounds for incorporation into polymer materials is ongoing, with anolyte-type compounds in high demand. In response to this need, we have synthesized and tested a range of new water-soluble redox-active s-tetrazine derivatives, including both low molecular weight compounds and polymers with different architectures. S-tetrazines are some of the smallest organic molecules that can undergo a reversible two-electron reduction in protic media, making them a promising candidate for anolyte applications. We have successfully modified linear polyacrylic acid and poly(N-isopropylacrylamide-co-acrylic acid) microgels with pendent 1,2,4,5-tetrazine groups. Electrochemical testing has shown that the new tetrazine-containing monomers and, importantly, the water-soluble redox polymers, both linear and microgel, demonstrate the chemical reversibility of the reduction process in an aqueous solution containing acetate buffer. This expands the range of water-soluble anodic materials suitable for water-based organic RFBs. The reduction potential value can be adjusted by changing the substituents in the tetrazine core. It is also worth noting that the choice of electrode material plays an important role in the kinetics of the tetrazine reaction: the use of carbon electrodes is particularly beneficial.

## 1. Introduction

Attempts to make batteries more environmentally friendly have led to an increased interest in organic redox-active materials, which can be used in both combined and all-organic devices. These organic molecules are cheaper and more sustainable, as their cost is not limited by the production or availability of rare elements [1]. The structural diversity of organic redox-active materials is very wide; it includes organosulfur derivatives [2,3,4], carbonyl compounds [5,6], free radical systems [7,8], viologens [9], and some others. Nevertheless, new tailor-made organic molecules with targeted electrochemical and physical properties (such as solubility, redox potential values, and the possibility of multi-electron transfer) are currently in high demand.

One of the promising and “greener” alternatives to Li-ion batteries, which currently occupy a significant part of the market, is redox-flow batteries (RFBs). RFBs have the unique feature of allowing for independent scaling up of their power (determined by the electrode surface area) and capacity (related to the amount of redox-active compounds in the tanks) [10]. From this point, RFBs, which are suitable for large-scale energy storage technologies, can be seen as a potential complement to Li-ion batteries.

RFBs using water-soluble organic redox-active materials are of particular interest [11]. They are safer and cheaper than their non-aqueous and inorganic counterparts and are in line with sustainable chemistry requirements. Commercial RFBs all use aqueous supporting electrolytes, highlighting the need for water-soluble redox-active materials. Despite some progress in this area [12,13], the search for new water-soluble organic redox-active materials remains a challenging task.

One of the main trends is the development of polymer-based redox-active materials for aqueous organic RFBs. Polymers with densely populated redox sites demonstrate high charge-transport and charge-storage capacities [14,15]. The incorporation of a redox unit into the polymer chain often improves cycling stability and helps overcome technical issues related to cell design. More importantly, it prevents the movement of redox species across the membrane, which can lead to severe capacity loss and make the cell more demanding on the type of membrane separating the anode and cathode compartments [16,17]. For example, the popular organic redox-active compound TEMPO (2,2,6,6-Tetramethylpiperidin-1-yl)oxyl), in its low molecular weight form, is prone to cross over even through the most expensive ion-selective membranes. Polymers, on the other hand, can be separated using relatively inexpensive size-exclusion (dialysis) membranes and microporous separators. There are several publications where this approach was used for ferrocene tetramer and a linear viologen polymer in an organic solvent [18], viologen and ferrocene polymers in an organic solvent [19], polymers embedded with TEMPO, and viologen groups in water [20]. For reviews, one can also see [13,21]. Also, the addition of a polymer moiety can enhance the solubility of the redox-active group in a targeted solvent and prevent precipitation. However, there are still a very limited number of groups available for inclusion in polymers: mainly TEMPO, viologens, and ferrocenes. Water-soluble anode-type compounds are particularly scarce [13].

Considering this, we focused on developing new types of water-soluble polymers that contain s-tetrazine (1,2,4,5-tetrazines) as a redox-active functional group. The s-tetrazines are one of the smallest organic molecules capable of reversible two-electron reduction in the protic media [22] and can be used as an anolyte. Among other important benefits of the s-tetrazines, high solubility and exceptional chemical stability in combination with rather rich chemistry, allowing targeted structural modification, should be mentioned. The examples of the application of tetrazines in energy conversion and storage systems are relatively rare. They have been used in combination with Li anode [23,24]. Recently, the first testing of the low-weight tetrazines in a lab-scale non-aqueous redox flow battery has been performed and gave promising results [25]. Application of the tetrazine-containing water-soluble polymers as the anolyte in aqueous RFBs has not been reported as yet.

From the structural point of view, both linear and branched (crosslinked) polymers can be considered as the carriers for redox-active groups. Most of the studies are focused on polymers with linear architecture [26,27,28,29] due to the variability of the synthetic procedures and ease of control of the properties of the material. Recently, a few works appeared that deal with more complicated polymer structures such as microgels, i.e., the weakly cross-linked submicron-size polymer meshes [30]. The branched systems possess a lower viscosity than the linear polymers of similar molecular weight [31], while the concentration of the redox-active centers can be as high as that for the linear polymer-based systems, which is favorable for use in RFBs. The examples of redox-active water-soluble microgels are rare; the microgels on the base of poly(hydroquinone) [32,33] and TEMPO [34,35] have been reported. Aqueous dispersions of such microgels can be treated as the new branch in a promising class of materials based on redox-active colloids (RACs) [36].

In this paper, we report the synthesis of various water-soluble s-tetrazine derivatives, which can be used for further integration into polymer chains. Two approaches were tested for the synthesis of redox-active polymers: polymerization of tetrazine-containing monomers and incorporation of tetrazine fragments into already prepared polymers; the latter one was successful yielding a new water-soluble redox polymer. Besides the polymer, the first example of a tetrazine-based microgel is reported. The results of electrochemical testing of new tetrazine-containing monomers and new water-soluble redox polymers will be discussed.

## 2. Experimental

### 2.1. Materials and Methods

#### 2.1.1. Materials

N-isporopylacrylamide (NIPAM—monomer, Sigma-Aldrich, St. Louis, MO, USA), N,N’-methylenebisacrylamide (BIS—crosslinking agent, Sigma-Aldrich, St. Louis, MO, USA), ammonium persulfate (APS—initiator, Sigma-Aldrich, St. Louis, MO, USA), V-50 (initiator, Wako Chemicals, Osaka, Japan), and sodium dodecylbenzenesulfonate (SDBS—surfactant, Sigma-Aldrich, St. Louis, MO, USA) were used as received. Acrylic acid (AA—monomer, Sigma-Aldrich, St. Louis, MO, USA) was purified by distillation. Water was purified using a Millipore Milli-Q system. N,N-dimethyl formamide (Acros Organics, Geel, Belgium) was purified by vacuum distillation with a 30 cm Vigreaux column over P_2_O_5_ (45–46 °C/12 Torr). Acetonitrile (Aldrich spectroscopic quality, <0.02% water content) was distilled over P_2_O_5_ and stored under argon. Acetone and hexane were distilled over CaCl_2_. Chloroform was distilled over P_2_O_5_. Other reagents were purchased from Aldrich and used without further purification.

Linear polyacrylic acid (PAA) with an average molecular weight M_n_ = 12.5 kDa and dispersity D = 1.3, synthesized via reversible addition-fragmentation chain-transfer (RAFT) polymerization using benzyl dithiobenzoate as a RAFT-agent [37], was received by the courtesy of Prof. Chernikova (Moscow State University, Russia) [38,39]. See SI for the synthetic and characterization details (text and Figure S23).

#### 2.1.2. Synthesis

Synthesis of 3-(3,5-dimethyl-1H-pyrazol-1-yl)-6-(2-ethoxyethoxy)-1,2,4,5-tetrazine (**2**)

A mixture of 3,6-bis(3,5-dimethyl-1H-pyrazol-1-yl)-1,2,4,5-tetrazine (0.3 g, 1.1 mmol), 2-ethoxyethan-1-ol (0.1 g, 1.1 mmol), triethylamine (0.34 g, 3.3 mmol), and acetonitrile (5 mL) was heated at 50 °C for 2 h. Then, the reaction mixture was evaporated under reduced pressure. The residue was separated with column chromatography on silica using a DCM:AcOEt 5:1 (*v*/*v*) mixture as an eluent. The targeted product (0.21 g, 72%) was obtained as a pink solid. ^1^H NMR (400 MHz, CDCl_3_) δ = 6.14 (1H, s, ArH), 4.83–4.79 (2H, m, CH_2_), 3.93–3.89 (2H, m, CH_2_), 3.60 (2H, q, *J* = 7.0 Hz, CH_2_), 2.63 (3H, s, CH_3_), 2.36 (3H, s, CH_3_), 1.22 (3H, t, *J* = 7.0 Hz, CH_3_). ^13^C NMR (101 MHz, CDCl_3_) δ = 166.27, 159.40, 153.57, 143.05, 111.16, 69.40, 68.01, 67.00, 15.15, 14.30, 13.85. ESI-HRMS: *m*/*z* 265.1411 ([M + H]^+^, 265.1408 calcd. for C_11_H_17_N_6_O_2_^+^). See Figures S1, S2 and S13.

Synthesis of 3,6-bis(2-ethoxyethoxy)-1,2,4,5-tetrazine (**3**)

A mixture of 3,6-bis(3,5-dimethyl-1H-pyrazol-1-yl)-1,2,4,5-tetrazine (1.0 g, 3.7 mmol), 2-ethoxyethan-1-ol (3.6 mL, 37 mmol), and triethylamine (0.77 mL, 5.5 mmol) was heated at 100 °C for half an hour. Then, the reaction mixture was evaporated under reduced pressure and the residue was separated with column chromatography on silica using a DCM:AcOEt 10:1 (*v*/*v*) mixture as an eluent. The targeted product (0.556 g, 58%) was obtained as a pink solid. ^1^H NMR (400 MHz, CDCl_3_) δ = 4.71–4.66 (m, 4H, m, CH_2_), 3.89–3.85 (4H, m, CH_2_), 3.59 (4H, q, *J* = 7.0 Hz, CH_2_), 1.21 (6H, t, *J* = 7.0 Hz, CH_3_). ^13^C NMR (101 MHz, CDCl_3_) δ = 166.22, 69.10, 68.18, 67.01, 15.20. ESI-HRMS: *m*/*z* 259.1399 ([M + H]^+^, 259.1401 calcd. for C_10_H_19_N_4_O_4_^+^). See Figures S3, S4 and S14.

Synthesis of 3-(2-aminoethylamine)-6-morpholino-1,2,4,5-tetrazine (**4**)

A mixture of 3,6-bis(3,5-dimethyl-1H-pyrazol-1-yl)-1,2,4,5-tetrazine (0.1 g, 0.4 mmol), morholine (42 mkL, 0.5 mmol), and acetonitrile (1.5 mL) was left for a night. Then, ethylendiamine (82 mkL, 1.2 mmol) was added. In an hour, the formed precipitate was filtered out and separated with column chromatography on silica using a CHCl_3_:MeOH 1:1 mixture as an eluent. The target compound (40 mg, 43%) was obtained as a reddish-pink solid. ^1^H NMR (400 MHz, CDCl_3_) δ = 5.76 (t, *J* = 5.9 Hz, 1H, NH), 3.85–3.80 (m, 4H, CH_2_), 3.78–3.73 (m, 4H, CH_2_), 3.55 (q, *J* = 5.9, 2H, CH_2_), 2.99 (t, *J* = 5.9 Hz, 2H, CH_2_), 1.58 (br s, 2H, NH_2_). ^13^C NMR (101 MHz, CDCl_3_) δ 160.90, 160.52, 66.57, 44.60, 44.34, 41.14. ESI-HRMS: *m*/*z* 226.1416 ([M + H]^+^, 226.1411 calcd. for C_8_H_16_N_7_O^+^). See Figures S5, S6 and S15.

Synthesis of 2-((6-(2-ethoxyethoxy)-1,2,4,5-tetrazin-3-yl)oxy)ethyl acrylate (**5**)

A mixture of 3,6-bis(2-ethoxyethoxy)-1,2,4,5-tetrazine (187 mg, 0.71 mmol), 2-hydroxyethyl acrylate (103 mg, 0.91 mmol), DBU (54 mg, 0.35 mmol), and DCM (1.5 mL) was heated for an hour. Then, the reaction mixture was separated with column chromatography on silica using DCM as an eluent. The targeted product (40 mg, 20%) was obtained as a pink solid. ^1^H NMR (400 MHz, CDCl_3_) δ = 6.43 (dd, *J* = 17.3, 1.4 Hz, 1H, C=CH), 6.13 (dd, *J* = 17.3, 10.4, 1H, C=CH), 5.86 (dd, *J* = 10.4, 1.4 Hz, 1H, C=CH), 4.82–4.79 (m, 2H, OCH_2_), 4.71–4.69 (m, 2H, OCH_2_), 4.64–4.57 (m, 2H, OCH_2_), 3.90–3.87 (m, 2H, OCH_2_), 3.59 (q, *J* = 7.0 Hz, 2H, CH_2_), 1.22 (t, *J* = 7.00 Hz, 3H, CH_3_). ^13^C NMR (101 MHz, CDCl3) δ 166.38, 165.99, 165.93, 131.85, 127.89, 69.22, 68.15, 67.38, 67.04, 61.97, 15.21. ESI-HRMS: *m*/*z* 285.1191 ([M + H]^+^, 285.1193 calcd. for C_11_H_17_N_4_O_5_^+^). See Figures S7, S8 and S16.

Synthesis of N-(3-((6-(2-ethoxyethoxy)-1,2,4,5-tetrazin-3-yl)amino)propyl)methacrylamide (**6**)

A mixture of 3,6-bis(2-ethoxyethoxy)-1,2,4,5-tetrazine (167 mg, 0.65 mmol), N-(3-aminopropyl)methacrylamide hydrochloride (100 mg, 0.56 mmol), triethylamine (72 mg, 0.71 mmol), and acetonitrile (1.5 mL) was left for two days. Then, the reaction mixture was separated with column chromatography on silica using a DCM:acetone 1:1 (*v*/*v*) mixture as an eluent. The targeted product (50 mg, 68%) was obtained as a red oil. ^1^H NMR (400 MHz, CDCl_3_) δ = 6.34 (1H, s, NH), 6.23 (1H, t, *J* = 6.6 Hz, NH), 5.77–5.74 (1H, m, C=CH), 5.37–5.35 (1H, m, C=CH), 4.65–4.57 (2H, m, OCH_2_), 3.88–3.81 (2H, m, OCH_2_), 3.64–3.55 (2H, m, NCH_2_), 3.44 (2H, q, *J* = 7.0 Hz, OCH_2_), 2.01–1.97 (m, 3H, CH_3_), 1.92–1.83 (2H, m, CH_2_), 1.22 (3H, t, *J* = 7.0 Hz, CH_3_). ^13^C NMR (101 MHz, CDCl_3_) δ = 169.13, 164.61, 161.99, 139.77, 120.00, 68.31, 68.13, 66.88, 38.59, 36.56, 29.14, 18.71, 15.16. ESI-HRMS: *m*/*z* 311.1827 ([M + H]^+^, 311.1826 calcd. for C_13_H_23_N_6_O_3_^+^). See Figures S9, S10 and S17.

Synthesis of 2-((6-(2-ethoxyethoxy)-1,2,4,5-tetrazin-3-yl)amino)ethan-1-ol (**7**)

A mixture of 3,6-bis(2-ethoxyethoxy)-1,2,4,5-tetrazine (0.3 g, 1.2 mmol), ethanolamine (100 mg, 1.6 mmol), and chloroform (3 mL) was left for four days. Then, the reaction mixture was separated with column chromatography on silica using a chloroform:acetone 1:1 (*v*/*v*) mixture as an eluent. The targeted product (50 mg, 83%) was obtained as a red oil. ^1^H NMR (400 MHz, CDCl_3_) δ = 6.56 (1H, t, *J* = 5.8 Hz, NH), 4.61–4.54 (2H, m, CH_2_), 3.88–3.80 (4H, m, CH_2_), 3.68–3.61 (2H, m, CH_2_), 3.57 (2H, q, *J* = 7.0 Hz, CH_2_), 3.42 (1H, br. s., OH), 1.19 (3H, t, *J* = 7.0 Hz, CH_3_). ^13^C NMR (101 MHz, CDCl_3_) δ = 164.52, 161.94, 68.30, 68.17, 66.93, 60.89, 44.04, 15.14. ESI-HRMS: *m*/*z* 230.1249 ([M + H]^+^, 230.1248 calcd. for C_8_H_16_N_5_O_3_^+^). See Figures S11, S12 and S18.

Synthesis of 2-((6-(2-ethoxyethoxy)-1,2,4,5-tetrazin-3-yl)amino)ethyl propionate (**9**)

A mixture of carbonyldiimidazole (39 mg, 0.24 mmol), propionic acid (20 mg, 0.27 mmol), and DMF (1 mL) was heated at 50 °C for an hour. Then, 2-((6-(2-ethoxyethoxy)-1,2,4,5-tetrazin-3-yl)amino)ethan-1-ol (**7**) (38.5 mg, 0.13 mmol) was added and the resulting mixture was heated at 70 °C for 48 h. The reaction mixture was poured into water (50 mL) and extracted with diethyl ether (3 × 10 mL). The combined extracts were dried over Na_2_SO_4_ and evaporated under reduced pressure. The residue was separated with column chromatography on silica using a chloroform:acetone 10:1 mixture as an eluent. The targeted product (21 mg, 55%) was obtained as a red oil. ^1^H NMR (400 MHz, CDCl_3_) δ = 5.79 (t, *J* = 5.72 Hz, 1H, NH), 4.64–4.60 (m, 2H, CH_2_), 4.33 (dd, *J* = 5.75, 4.82 Hz, 2H, CH_2_), 3.88–3.84 (m, 2H, CH_2_), 3.82 (td, *J* = 5.97, 5.14 Hz, 2H, CH_2_), 3.60 (q, *J* = 7.0 Hz, 2H, CH_2_), 2.36 (q, *J* = 7.5 Hz, 2H, CH_2_), 1.22 (t, *J* = 7.0 Hz, 3H, CH_3_), 1.14 (t, *J* = 7.5 Hz, 3H, CH_3_). ^13^C NMR (101 MHz, CDCl_3_) δ 174.59, 165.09, 161.76, 68.37 (2C), 67.01, 62.52, 41.02, 27.54, 15.25, 9.15. ESI-HRMS: *m*/*z* 286.1516 ([M + H]^+^, 286.1510 calcd. for C_11_H_20_N_5_O_4_^+^).

The copolymerization of NIPAM and tetrazines with the attached acrylate group (compounds **5** and **6** at Scheme 2) was performed using free-radical precipitation polymerization. In total, 0.5 g of NIPAM, 0.05 g of tetrazine monomer, 0.01 g of BIS, and 0.01 g of initiator (either APS or V-50) were dissolved in 50 mL of deionized water. Polymerization was carried out in a glass reactor in an argon atmosphere at a temperature of 80 °C under continuous stirring at a rate of 800 rpm for 20 h. The resulting solution was purified from reaction by-products by dialysis against deionized water (12 kDa MWCO) for 7 days.

PNIPAM-PAA microgels were synthesized by surfactant-assisted precipitation polymerization in water [40]. Polymerization was carried out under an argon atmosphere in a glass reactor equipped with a thermostatically controlled chamber and a magnetic stirrer. In a typical procedure, 1.6 g of NIPAM, 0.44 g of AA, 0.03 g of BIS, and 0.025 g of SDBS were dissolved in 50 mL of water and placed into the reaction vessel. The solution was stirred (800 rpm) at 80 °C for 60 min under the argon flow to allow complete dilution and oxygen removal. Then, the APS initiator was added to start the reaction. The polymerization was carried out for 20 h; then, the solution was purified by dialysis against deionized water (12 kDa MWCO) for 7 days. According to element analysis, the rate of C:N:H was 57.85:9.20:8.92.

#### 2.1.3. Modification of the Polymers with Tetrazines

A mixture of the polymer, either linear PAA or microgels (13.2 mg), carbonyldiimidazole (9.6 mg, 0.06 mmol), 2-((6-(2-ethoxyethoxy)-1,2,4,5-tetrazin-3-yl)amino)ethan-1-ol (**7**) (31.8 mg, 0.14 mmol), and DMF (2 mL), was heated at 70 °C for 20 h. Then, the solvent exchange from DMF to water proceeded by the following procedure. The solution was poured into an appropriate dialysis bag (MWCO 3.5 kDa, Servapor dialysis tubing), and then the bag was placed in a large amount of deionized water. The system was left to equilibrate for one week with regular replacement of the outer solution. After that, the solution was filtered by a PVDF hydrophilic syringe filter, 0.45 μm, Labfi, to remove the residual water-insoluble chemicals.

#### 2.1.4. Methods

*Dynamic light scattering* (DLS) measurements were performed by a static/dynamic compact goniometer (DLS/SLS-5000, ALV, Langen, Germany). A HeNe laser with a power of 22 mW emitting a polarized light at λ = 633 nm was used as the incident beam. DLS measurements were taken at 23 °C at the scattering angle of 90°. The mass concentration of the samples was 0.3 g/L. Distributions over decay time τ were obtained by means of a nonlinear regularized inverse Laplace transformation method (CONTIN) [41]. Apparent self-diffusion coefficients D were determined from the angular dependence of the relaxation time τ in accordance with D = 1/τq^2^ the relation, where q = (4πn/λ)sin(θ/2) is the wave vector magnitude. The corresponding hydrodynamic radii R_h_ were calculated from the Stokes–Einstein equation, R_h_ = kT/6πηD, where k is Boltzmann’s constant and η is the solvent viscosity.

Atomic force microscopy (AFM) imaging was performed using a MultiMode IIIa scanning probe microscope (Bruker, Billerica, MA, USA) in tapping mode. Silicon cantilevers (Bruker, USA) with a resonant frequency of around 300 kHz and a tip radius of around 10 nm were used. Image processing and presentation were conducted by means of FemtoScan Online© SPM Image processing software (Advanced Technologies Center, Moscow, Russia). A drop of the sample aqueous solution was deposited onto the silicon wafer plate and left for drying.

ESI-MS spectra were registered by Sciex TripleTOF 5600+. Acetonitrile was used as a solvent. Measurements were performed in positive ion mode.

Mass spectrometry analysis was performed using gas chromatograph Agilent 8890 with the mass selective detector 5977B Inert Plus MSD Turbo EI Bundle. Electron impact ionization with the 70 eV energy of electrons was applied. The liner temperature was 300 °C. The temperature regime of columns was as follows: 4 min at 70 °C, linear heating up to 300 °C by the 13th minute, and then keeping at 300 °C.

NMR spectra were recorded using Bruker Avance 400. Chemical shifts were referenced to a residual non-deuterated solvent.

UV–Vis spectra in the range 200–1100 nm were recorded using AvaSpec Avantes spectrometer.

Electrochemical characterization was performed using a Biologic BP-300 potentiostat (Seyssinet-Pariset, France) in an ALS Co. three-electrode cell (electrolyte volume ~2 mL) with a platinum wire counter electrode and an Ag/AgCl/KCl_sat._ reference electrode. Pt and glassy carbon disk electrodes with an active surface area of 0.020 and 0.070 cm² were used as working electrodes. A hardware ohmic drop compensation was employed. The resistance between the reference and the working electrodes was measured using the EIS spectra. All solutions were thoroughly deaerated by passing an argon flow through the solution prior to the experiments and above the solution during the measurements. Aqueous acetate buffer solutions (0.2 M AcONa and acetic acid to pH 4.4 and 3.5) were used as electrolytes.

Investigation of long-term electrochemical stability was performed using a Biologic SP-300 potentiostat in a custom-made three-electrode cell (electrolyte volume ~3 mL) with a platinum wire counter electrode and an Ag/AgCl/KCl_sat._ reference electrode. Carbon paper Toray TGP-H-90 (pristine and annealed for 30 h at 400 °C under air) was used as a working electrode. All solutions were thoroughly deaerated by passing an argon flow through the solution. An aqueous acetate buffer solution (pH 4.4) was used as a supporting electrolyte.

## 3. Results and Discussion

### 3.1. Synthesis of the Water-Soluble Low-Weight s-Tetrazine Derivatives

**Scheme 1:**
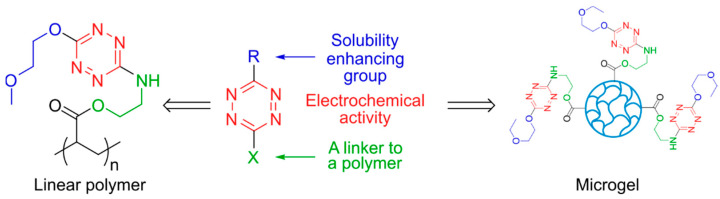
Schematic illustration of tetrazine functional groups and the pathways for its incorporation in polymer moiety.

Our first goal was to synthesize the water-soluble form of the s-tetrazine molecule and equip it with the functional group responsible for the incorporation in the polymer chain (Scheme 1).

**Scheme 2:**
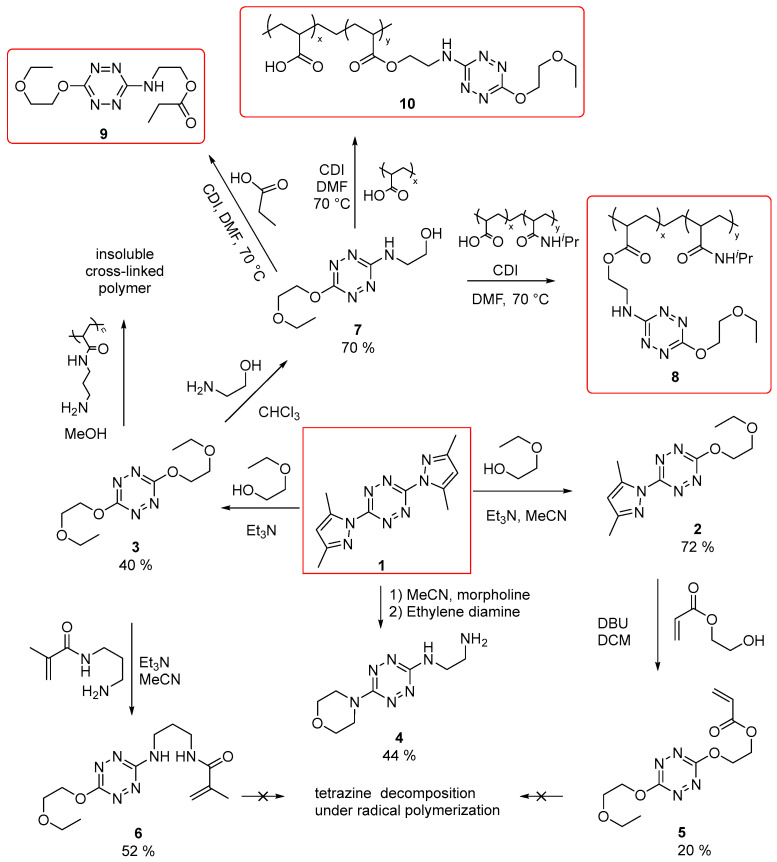
Synthesis of the tetrazine derivatives.

Tetrazines containing the leaving groups in 3,6 positions are prone to nucleophilic aromatic substitution, which provides a convenient route to their structural modification [42]. Easily available 3,6-dipyrazolyltetrazine **1** was used as the starting compound (Scheme 2). The presence of two leaving groups was required for the stepwise functionalization of the tetrazine core with the hydrophilicity enhancing moiety (unsubstituted tetrazines are only very weakly soluble in water) as well as with the other structural motif responsible for the follow-up formation of the redox-active polymer. This synthetic strategy was used for the synthesis of the tetrazine-containing monomers as well as for the grafting of the tetrazine moiety into the already prepared polymer.

The ethylcellosolve and morpholine fragments were chosen as the substituents increasing the hydrophilicity of the tetrazines. The reaction of **1** with ethyl cellosolve was carried out in mild conditions at room temperature in the acetonitrile solution to prevent the substitution of both pyrazole rings. The targeted compound **2** was isolated in 72% yield. If the reaction is performed using ethyl cellosolve as the solvent, both pyrazole rings are replaced with the ethyl cellosolve fragments (**3**). The replacement of the pyrazole ring for the morpholine fragment yielded tetrazine **4** in 44%. Unexpectedly, the morpholine-containing tetrazine **4** turned out to be poorly soluble in water. Consequently, it is a not perspective for the microgel formation and further research was performed with the tetrazines modified with the ethyl cellosolve group.

There are two possible ways for the incorporation of the tetrazine fragments into the polymer chain: (1) formation of the tetrazine-containing monomer capable of further (co)polymerization and (2) synthesis of the functionalized low molecular weight tetrazine suitable for grafting on the pre-prepared polymer (preferably, by easy click-chemistry-like reaction).

Within the first approach, compounds **2** and **3** were used for the synthesis of the acrylate-containing monomers **5** and **6**. The reaction of **2** with 2-hydroxyethyl acrylate gave the targeted mono-acrylate derivative a low yield (20%) due to the competing replacement of both leaving groups for the 2-hydroxyethyl acrylate. Additionally, a significant amount of tetrazine **3** containing two ethyl cellosolve groups was also present in the reaction mixture decreasing the yield of the targeted derivative **5**. It was shown that the nucleophilic substitution in tetrazines is a dynamic and reversible process: the presence of two different leaving groups and an external nucleophile may yield a complicated mixture of the products [43]. The exchange would be selective only if one of the reagents is a much better leaving group. Meanwhile, that was not the case for the combination of pyrazole and ethyl cellosolve groups. Additionally, the leaving group abilities of the ethyl cellosolve and 2-hydroxyethyl acrylate fragments are also similar, yielding the inseparable mixture with a low content of the targeted monomer **5**.

To overcome the problem, an alternative approach to the insertion of the polymerizable acrylate fragment was also tested, taking **3** as the starting compound. The nucleophilicity of N-3-aminopropyl methacrylamide is higher and it forms a poor leaving group as compared to 2-hydroxyethyl acrylate. The reaction with the methacrylamide derivative was successfully performed in mild conditions: the targeted tetrazine **6** with a polymerizable acrylate group was isolated in a practical 52% yield.

An alternative route to the tetrazine-containing polymers was also tested, based on the insertion of the redox-active moiety in the already prepared polymer chain. The reaction of **3** with the poly(acrylamide) containing 3-aminopropyl pendant groups gave a cross-linked polymer soluble neither in water nor in organic solvents. More likely, the substitution of both ethyl cellosolve groups takes place. To overcome the problem, **3** was converted to derivative **7** (70%) with the N-containing poor leaving group. Thus, modified s-tetrazine **7** was used as a redox-active unit to be further grafted to the carboxylic groups of the chosen polymers. For the comparison, the model low-weight tetrazine derivative **9** was also prepared. All new tetrazine derivatives **2**–**7** and **9** were completely characterized using HRMS and NMR spectroscopy (Figures S1–S18 in Supplementary Materials). The ^13^C NMR spectra of all compounds contain the signals in the 160–166 ppm range inherent to the tetrazine carbon atoms [44] (see Supplementary Materials for the details).

### 3.2. Preparation and Characterization of the Water-Soluble Polymers Decorated with Tetrazine Groups

#### 3.2.1. Polymerization of the Tetrazine Monomers

The acrylate-containing monomers **5** and **6** were subjected to the free-radical copolymerization with the N-isopropylacrylamide monomers (see Experimental section for the details) to synthesize small cross-linked polymer particles, the microgels [45]. Interestingly, the characteristic orange color indicating the presence of the tetrazine fragment has completely disappeared during the synthesis, indicating its destruction. The control experiments with monomer **7** showed that the characteristic absorption band at 417 nm inherent to the tetrazine unit completely disappears after half an hour heating (80 °C) in the presence of the polymerization initiator (K_2_S_2_O_8_), see Figure S20 in Supplementary Materials. A plausible mechanism of decomposition may involve the addition of the sulphate radicals to the tetrazine units. Thus, the commonly used protocol for the radical polymerization [46] turned out to be inapplicable in our case. Moreover, it seems that any type of radical polymerization (including polymerization in bulk under vacuum or the use of controlled polymerization methods) will lead to the decomposition of the tetrazine fragments due to the presence of active radicals. Thus, we had to abandon this strategy of the tetrazine-containing polymer preparation and concentrated on the grafting approach.

#### 3.2.2. Water Soluble Linear PAA with the s-Tetrazine Pending Groups (**10**)

The modification of a polymer with pendant carboxylic groups using the tetrazine-containing alcohol **7** can be performed via the esterification process. First, the experimental procedure was optimized using propionic acid as a model. Activation of the carboxy groups with carbonyldiimidazole (CDI) [47] was performed. However, the follow-up reaction with tetrazine-containing alcohol **7** in the presence of a strong base (1,8-diazabicyclo(5.4.0)undec-7-ene, DBU) resulted in the partial decomposition of the tetrazine moiety. Since the preliminary experiments showed that the tetrazine moiety did not withstand the presence of a base, it was excluded, whereas the reaction temperature was increased (from 50 to 70 °C) as well as the reaction time. Thus, the optimized procedure was applied to the modification of the polymers.

**Figure 1:**
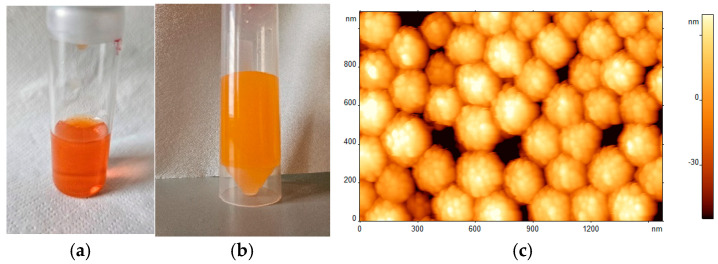
The photos of (**a**) PAA-TZ and (**b**) MG-TZ dispersions in water; (**c**) AFM-image of the dried drop of MG-TZ dispersion.

In the first experiment, the s-tetrazine pending groups from **7** were used for the decoration of the linear polymer containing carboxylic groups, i.e., the polyacrylic acid (PAA) with M_w_ = 12,500. The resulting solution was thoroughly purified by dialysis and filtering to remove the residual chemicals. The aqueous solution of the tetrazine-modified PAA (PAA-TZ) **10** turned out to be transparent; its bright orange color was typical for the tetrazine groups (See Figure 1a).

A dynamic light scattering (DLS) investigation was performed to study the behavior of the polymer before and after modification in both acidic and basic conditions. PAA is known to be a pH-sensitive polymer; the degree of its monomer ionization, and, consequently, the size of a polymer coil, is dependent on the solution pH with pKa = 4.25 [48]. Table 1 summarizes the results; the size distributions were very narrow in all cases (Figure S21 in Supplementary Materials). It is clearly seen that the modified polymer **10** possesses a much bigger hydrodynamic radius R_h_ compared to the starting one, both below and above the pKa of acrylic acid. That may be due to either the substantial increase in its effective hydrodynamic radius (as a result of the attachment of many bulky TZ pending groups forming the “brush-like” structure) or the partial aggregation of the chains into stable nanosized aggregates. PAA-TZ did not precipitate in an aqueous medium; the solution remained homogeneous for several weeks.

#### 3.2.3. Hydrophilic s-Tetrazine-Modified Poly(N-isopropylacrylamide-co-acrylic acid) Microgel (**8**)

The cross-linked polymer particles, namely poly(N-isopropylacrylamide-co-acrylic acid) microgels (MG), with M_w_ = 1.7 × 10^9^ Da molecular weight, 27 mol% of AA [40], and hydrodynamic radius R_h_ equal to 350 nm at pH 6.5 and T = 23 °C (Table 1), were also tested as the possible s-tetrazine carriers. The initial microgel particles can be easily prepared via the one-step synthesis using free-radical precipitation polymerization; these microgels are known to form stable dispersions in aqueous conditions [49].

The microgels with grafted tetrazine groups (MG-TZ) retained the ability to form dispersions in water without aggregation and precipitation in a wide range of conditions, producing a semi-opaque liquid with a typical tetrazine orange hue. The dispersion image and the AFM image of the dried MG-TZ are presented in Figure 1c. The distinct spherical monodisperse microgel particles are clearly seen in the AFM microphotograph.

In detail, the properties of MG-TZ dispersions were analyzed using dynamic and static light scattering methods (DLS/SLS). We found that MG-TZ typically appears to be spheres with R_h_ ~ 120 nm in water, indicating that the grafting process did not lead to the inter-crosslinking of the gel particles (Table 1). The significant reduction in the size of the MG-TZ compared to the initial microgel size can be attributed to the reduction in the number of carboxylic groups during the modification process. The shape factor of the MG-TZ particles (R_g_/R_h_ value where R_g_ is the radius of gyration) is inherent to the collapsed microgels, corresponding to a homogeneous sphere shape factor. It was found that the MG-TZ aqueous dispersions are stable in a wide pH range (from ~4.5 to 10) at all the temperatures measured (See Figure S22 in Supplementary Materials). At low pH, the microgels form aggregates and gradually precipitate with time; the effect is promoted with the temperature increase. Here, it should be noted that the initial microgels are endowed by both thermo- and pH-sensitivity due to the nature of the comprising polymers [50,51].

**Figure 2:**
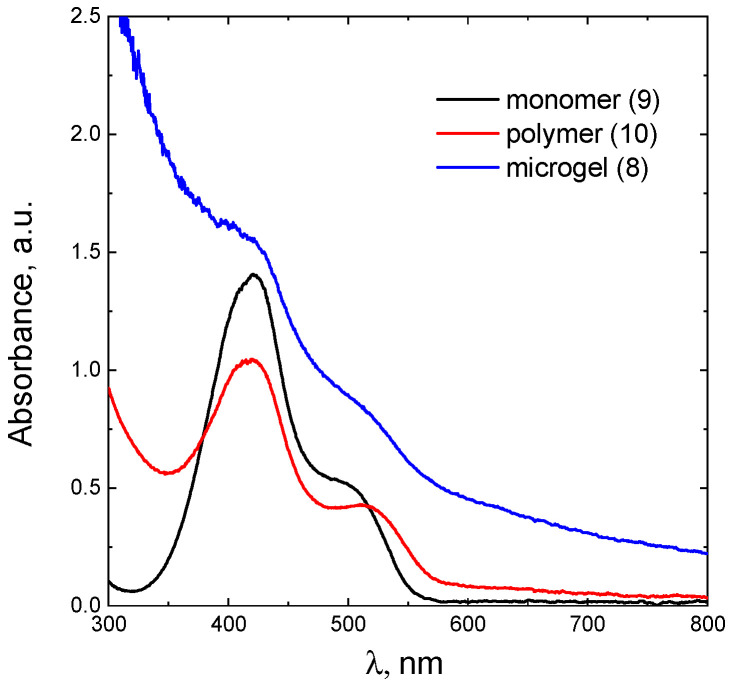
UV–vis spectra of **8**–**10** in water solution.

To confirm that the tetrazine units were successfully grafted into the polymer matrix, a comparative study of the electronic spectra of the low-weight derivative **9** as well as polymers **8** and **10** was performed. As follows from Figure 2, the characteristic absorbance at 417 and 503 nm, typical for the tetrazine moiety [52], was observed for **8**–**10** in aqueous solution. That signifies that the process of the Tz-groups attachment to both the linear polymer chain and the cross-linked microgel species proceeded successfully. In the case of microgel **8** and polymer **10**, the tetrazine’s absorbance is superimposed to the smooth downward curve corresponding to the light scattering. This was taken into account as background and the absorption at 417 nm was determined. Assuming that the molar absorption of the tetrazine group is not affected by the binding to the polymer, the molar absorption for tetrazine **7** (see Figure S19) was used to determine the concentration of the tetrazine fragments in the modified polymers **8** and **10** and the degree of modification. It was shown that 10 and 3% of the monomer units were modified with the tetrazine moiety in **10** and **8**, respectively. The lower grafting in the microgel is a consequence of higher sterical blocking of the carboxylic groups. It should be additionally emphasized that the samples were extensively purified by dialysis and filtration prior to all experiments, assuming the absence of non-bonded tetrazines.

The polymer and microgel compounds under study swell in water at room temperature without precipitation since it is a good solvent for them. The viscosity of the polymer (or polymer microgel) solution increases with the concentration increase due to the formation of the entanglements between polymer chains. In the case of our species, the apparent viscosity increases sharply at concentrations above 3 wt%, which means that it passes from diluted to semi-diluted mode [53,54]. Tetrazine **7** is hygroscopic; it dissolves in very small quantities of water. Since, in redox flow batteries, the reagents are pumped through the electrochemical cell, meaning they should not be too viscous, this concentration value can be taken as a solubility threshold.

### 3.3. Electrochemical Properties of the Water-Soluble Tetrazine Derivatives

#### 3.3.1. Redox-Activity of the Low-Weight Tetrazines in Aqueous Solution

**Scheme 3:**
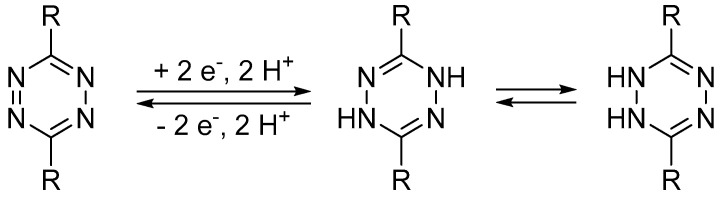
Typical redox process of s-tetrazine.

To test the suitability of new water-soluble tetrazine derivatives as the redox active pendant groups in polymers, with a further focus on use in energy conversion and storage area, their voltammetric behavior was investigated. Notably, electrochemical properties of s-tetrazines in organic solvents have been widely explored (see, e.g., refs. in the review [52]). Thus, a reversible one-electron reduction at around −1.25–1.55 V vs. Fc^+^/Fc can be observed for the tetrazines substituted with the NR_2_ and OR groups that are structurally similar to the derivatives described herein [52]. In contrast, the data on the tetrazine behavior in aqueous solutions are scarce, due to the limited solubility of the pristine s-tetrazine in water [22]. It was shown that the tetrazine fragment undergoes a two-electron two-proton one-step redox process at a potential of around −0.2 V vs. Ag/AgCl/KCl_sat._ at pH 7 in an aqueous solution [22] (Scheme 3).

**Figure 3:**
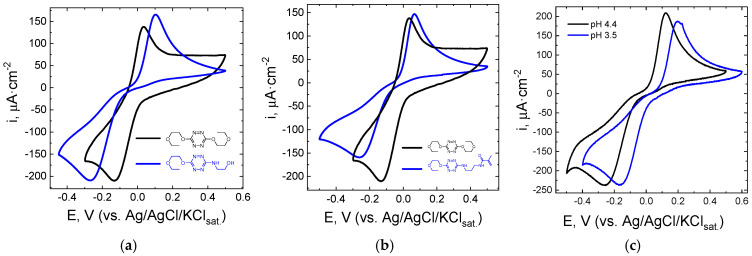
Cyclic voltammograms of **3** (1 mM) and **7** (1 mM) (**a**), **3** (1 mM) and **6** (1 mM) (**b**), (0.2 M AcONa and AcOH to pH 4.4, Pt, 25 mV/s); cyclic voltammograms of **3** (1 mM) (**c**), (0.2 M AcONa and AcOH to pH 4.4 and 3.5, Pt, 25 mV/s).

For energy conversion systems, two key points are important, i.e., the reversibility of the process and the electrochemical reaction rate. As follows from Scheme 3, the tetrazine reduction is coupled with the protonation. Thus, the buffering properties of the solution are important to accelerate the electrochemical reaction and to provide reversibility of the redox process. The voltametric curves measured for tetrazines **3**, **6**, and **7** are given in Figure 3a,b. One can see that the reduction is quasi-reversible (the peak currents ratio is close to unity), but the direct-reverse peak separation exceeds 160 mV even in the acetate buffer. The comparison of **3**–**7** and **3**–**6** couples showed that the electron-donating and bulky substituents slow down the electrochemical reaction. In contrast, an increase in the acidity of the medium (from pH 4.4 to 3.5) slightly increased the electrochemical reaction rate (Figure 3c).

The potential values measured for tetrazines **3**, **6**, **7**, and **9** at a Pt electrode in an acetate buffer solution are given in Table 2. Varying the substituents in **3** and **6** positions allows tuning of the potential values as well as increasing the rate of the redox process.

**Figure 4:**
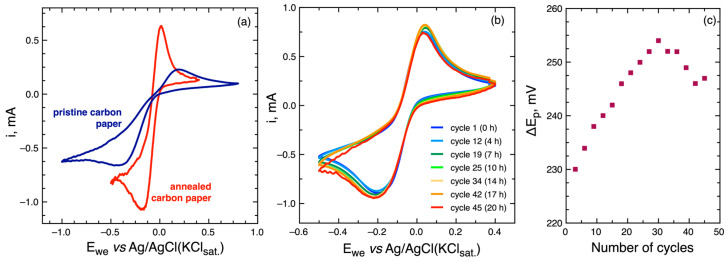
(**a**) Cyclic voltammograms of **7** at the pristine and annealed carbon paper electrodes in the 0.2 M acetate buffer solution at pH 4.4 (5 mV/s scan rate); (**b**) Cyclic voltammograms of **7** in a 0.2 M acetic buffer solution at pH 4.4 at annealed carbon paper electrode at a potential scan rate of 5 mV/s recorded over a time period of 20 h; (**c**) the evolution of cathodic peak current with time during repetitive cycling of tetrazine 7 on the annealed carbon paper electrode. The concentration of tetrazine moieties was 0.1 mM.

Electron transfer (ET) kinetics are known to be influenced by the working electrode material. In search of a way to improve the ET kinetics of terazine reduction, various electrode materials were tested. The application of a glassy carbon electrode taking tetrazine **7** as a model did not improve the situation (Figure S24): relatively slow kinetics (peak-to-peak separation ~ 400 mV) were observed. Afterward, a carbon paper electrode (Toray TGP-H-90), one of the common electrodes for flow cell studies [55,56], was tested. As has been previously shown, carbon electrodes preliminary annealed in air demonstrated enhanced performance [57,58] in a flow cell; therefore, cyclic voltammetry responses of tetrazine **7** on pristine and annealed (400 °C, 30 h) carbon paper were compared (Figure 4a). A slow scan rate (5 mV/s) was used to reduce the capacitive currents due to the high surface area of the electrode.

Indeed, reaction kinetics and reversibility of tetrazine **7** reduction were significantly enhanced (Figure 4a) as compared to the results obtained at the pristine electrode. Tetrazine **7** at the pristine electrode demonstrates higher peak-to-peak separation, i.e., slower reaction rate and lower reversibility. The possible reason can be a higher amount of oxygen groups formed on the carbon paper surface upon annealing in air. They may enhance the electrode process by participating in protonation steps involved in the tetrazine reduction reaction.

The long-term cycling of tetrazine **7** on the annealed carbon paper electrode was also performed. Groups of three consecutive cycles, each group followed by an OCP period of 1 h, were measured for 20 h. It can be seen that tetrazine **7** demonstrates decent cycling stability (Figure 4b). The peak-to-peak separation increases during the first 30 cycles and then there is a stabilization (and even reduction) of Δ*E*_p_ (Figure 4c). The change in peak-to-peak separation may be attributed to slow changes in the surface conditions of carbon paper upon cycling in acetate buffer solution.

Thus, the proper selection of the working electrode material allows the significant improvement in the new tetrazine reduction kinetics. Though their application in fast-operating systems such as electrochemical capacitors is still under question, these compounds may be considered of potential interest as possible anolyte reagents for RFBs.

#### 3.3.2. Electrochemical Characterization of the Tetrazine-Containing Polymers and Microgels

**Figure 5:**
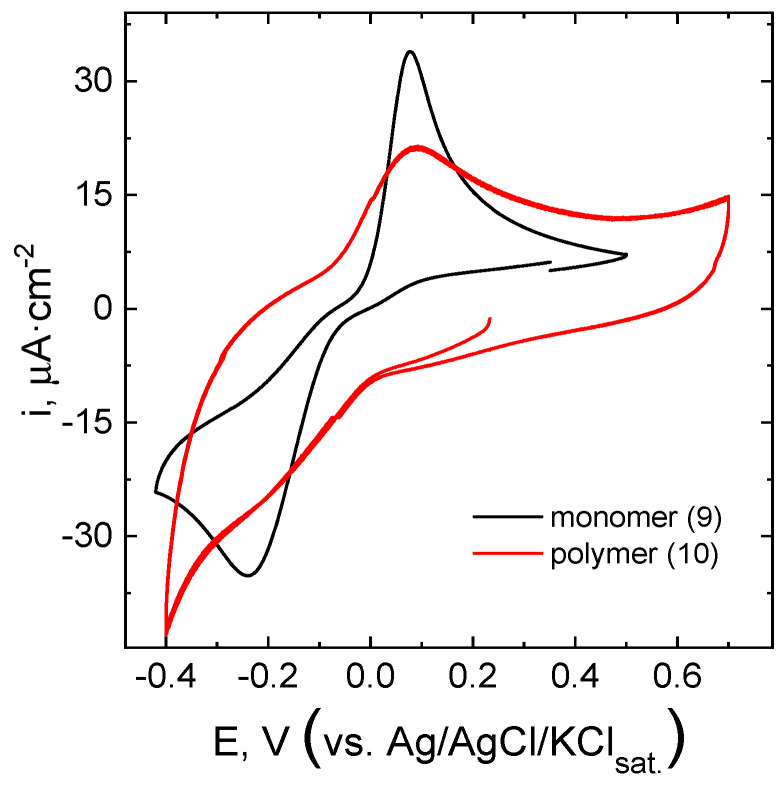
Cyclic voltammograms of monomer **9** (1 mM) and polymer **10** were measured on a glassy carbon electrode in 0.2 M acetate buffer solution (AcONa+AcOH, pH 4.4), scan rate 100.

**Figure 6:**
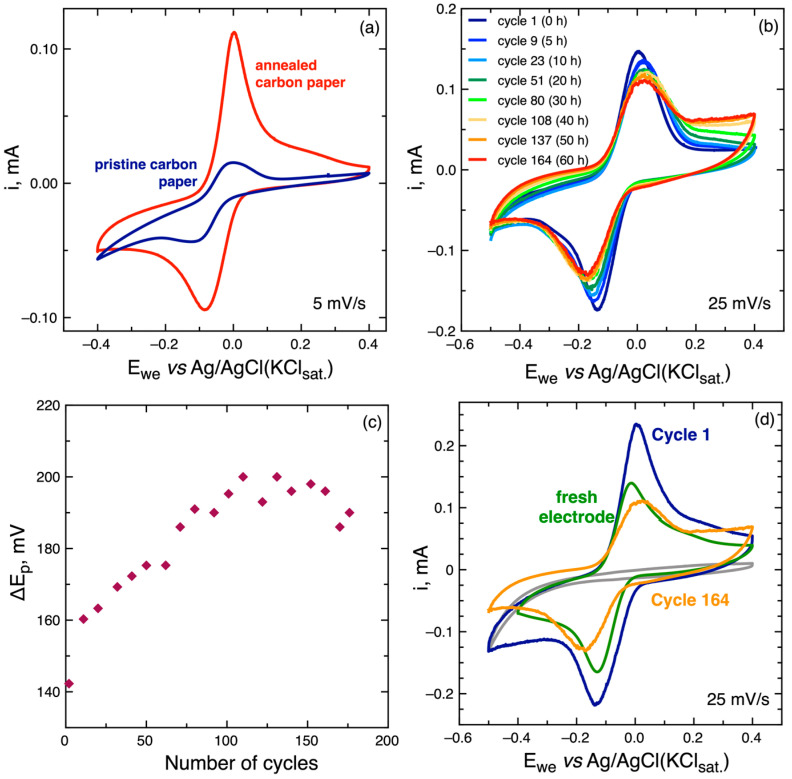
(**a**) Cyclic voltammograms of polymer **10** at the pristine and annealed carbon paper electrodes in the 0.1 M acetate buffer solution at pH 4.4 (5 mV/s scan rate); (**b**) Cyclic voltammograms of polymer **10** in 0.1 M acetic buffer solution at pH 4.4 at annealed carbon paper electrode at a scan rate of 25 mV/s, recorded over a time period of 60 h; (**c**) the evolution of peak-to-peak separation with time during repetitive cycling of polymer **10** on the annealed carbon paper electrode. (**d**) The 1st (blue) and 164th (red) cyclic voltammogram recorded on the annealed carbon paper electrode, and cyclic voltammogram (green) of a fresh carbon paper electrode in the same polymer **10** solution after long-term cycling. Grey curve represents the background voltammogram. The concentration of tetrazine moieties was 0.1 mM.

To estimate the suitability of polymers **8** and **10** for energy storage applications, their redox activity in aqueous solution was studied using cyclic voltammetry. The voltammetry curves measured for the linear polymer **10** and for its low-weight analog **9** on the glassy carbon electrode are given in Figure 5. One can see that the direct and reverse peak potential values for **9** and **10** coincide well. The direct-reverse peak separation is rather significant in both cases; this indicates that the electrochemical reaction is relatively slow. However, the voltammograms measured for polymer **10** at the annealed carbon paper electrode demonstrated good reversibility and much lower peak separation (~140 mV) in comparison with pristine carbon paper (Figure 6a), similar to what has been observed in the case of tetrazine **7**.

During the long-term cycling (Figure 6b, cyclic voltammograms were recorded by groups of three cycles, each group followed by an OCP period of 1 h), a steady increase in peak-to-peak separation (Figure 6c) was observed up to 100 cycles, and after that, there is stabilization at ~200 mV, i.e., polymer **10** demonstrated quite slow but relatively stable reaction kinetics.

To determine whether the increase in peak-to-peak separation is caused by surface changes in the carbon paper electrode or degradation of the polymer solution, the carbon paper electrode was removed from the cell after long-term cycling and replaced with a fresh electrode. We observed that peak-to-peak separation on the fresh electrode returned to its initial value (Figure 6d). Thus, its increase is attributed to the change in carbon paper surface conditions rather than the degradation of the polymer solution. The reduction currents observed in the background voltammogram (Figure 6d) at the potentials less than –0.3 V vs. Ag/AgCl may correspond to the electrochemical reduction in oxygen-containing functional groups on the carbon electrode surface, which may affect the reaction kinetics. The overall current decrease after changing the electrode is most probably a result of a change in the electrode surface area, as in the cell arrangement employed for the measurements, it was not possible to precisely control the carbon paper area immersed in the electrolyte.

Apparent self-diffusion coefficients D for the tetrazine-modified polymers and microgels were evaluated using the dynamic light method (see Materials and Methods section for the details). They were found to be equal to 6.7 × 10^−8^ cm^2^/s for PAA-TZ and 1.8 × 10^−8^ cm^2^/s for MG-TZ in water at pH 6.5, which is typical for such systems [35]. Determination of the ET rate constant is challenging since the electrochemical reduction of tetrazines is an ECEC process including consecutive electrochemical (E) and chemical (C) steps [22]. The classical Nicolson method [59] is not applicable in this case. Theoretically, the desired value could be obtained by computational modeling of CV series at different potential sweep rates. However, this is a complicated task since many independent parameters (two electron transfer constants, two reduction potentials, rates and equilibrium constants for two chemical steps, diffusion coefficient) must be fitted.

**Figure 7:**
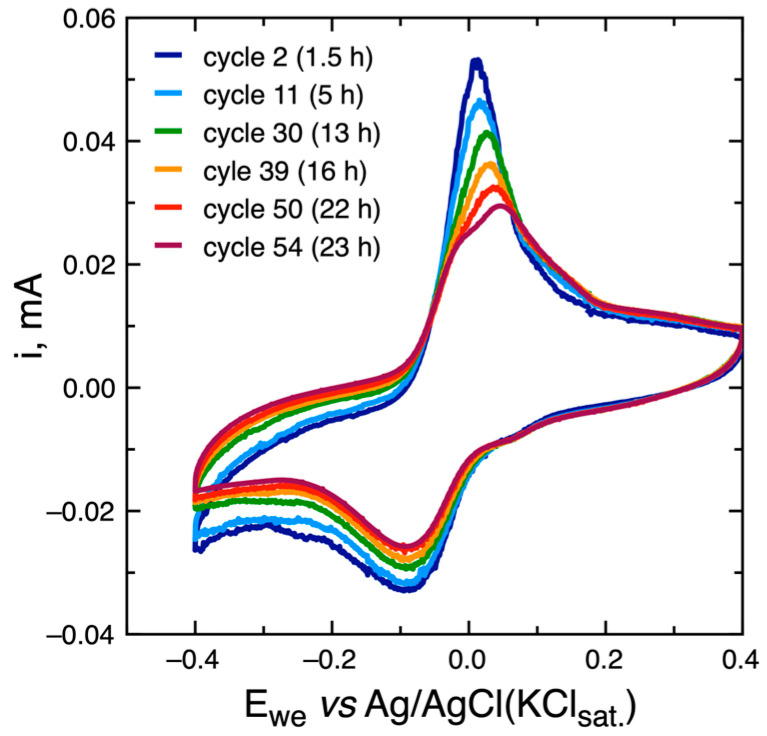
Cyclic voltammograms of microgel **8** in 0.2 M acetate buffer solution (pH 4.4) at the annealed carbon paper electrode were recorded over a time period of 23 h (the scan rate is 5 mV/s). The concentration of tetrazine moieties was 0.1 mM.

The slow protonation step might be a possible reason why we were unable to detect the redox activity of the microgel **8** on the glassy carbon electrode, despite the fact that it contains tetrazine fragments, as confirmed by UV–Vis data. However, we were able to observe the electrochemical response of microgel **8** on the annealed carbon paper electrode (Figure 7). The voltammograms demonstrate a prominent adsorption peak of reduced tetrazine fragments, which gradually decreases upon long-term cycling. Similar to the case of tetrazine-grafted linear polymers, it is probably caused by the reduction of oxygen-containing groups on the carbon electrode surface.

Slow in itself, the electrochemical reaction in microgels becomes even more impeded. The “network mesh structure” and relatively large size of the cross-linked microgel impede penetration of the protons required for the reduction. Based on these experimental results, we can speculate that the use of relatively small branched star polymers [60], or hyperbranched polymers [61], could be more effective carriers for these redox-active species, both from the point of the ET kinetics and the choice of separator membrane for the RFB cell. This study is in progress now. At present, the tetrazine-modified polymer **10** with well-pronounced redox activity may be considered the first step toward fabrication of a novel type of water-soluble anodic material.

The theoretical capacity of the presented species can be estimated as 234 mAh·g^−1^ for low molecular weight tetrazine **7** and as 58 mAh·g^−1^ for the linear polymer compound and 173 mAh·L^−1^ for its aqueous solution, taking into account the solubility and viscosity increase. However, there is still room for improvement, as only a small number of carboxylic groups have been modified with tetrazine units.

It would be interesting to compare new tetrazine derivatives with the previously reported electrolyte materials. Notably, the number of water-soluble organic molecules suitable for performance as anodic active materials is still rather limited (see reviews [12,13,62]). The most investigated species are antraquinones. Anthraquinone-2-sulfonic acid demonstrates a one-electron redox process at E^0^ = −0.07 vs. AgCl/Ag [63]); reduction of 3,4-dihydroxy-9,10-anthraquinone-2-sulfonic acid is a two-electron process with E^0^ = −0.12 V vs. AgCl/Ag [64]. Thus, the reduction potentials for antraquinones and tetrazines are similar; whereas, with regard to water solubility, the tetrazines have advantages over antraquinones. Methyl viologen derivatives can be considered as the other popular candidate for redox-active anodic material. They have higher water solubility as compared to tetrazines and show more cathodic reduction potential [65]. Unfortunately, they are toxic to human beings and animals and are banned in many countries. Their redox activity produces superoxide anions, which are linked to the development of Parkinson’s disease [66]. Also, naphthalene diimides demonstrate promising negolyte qualities but as low molecular weight compounds [67]. Thus, the search for the optimal water-soluble anodic material is still a challenge, and tetrazine derivatives may be considered as prospective ones.

## 4. Conclusions

In conclusion, a variety of water-soluble tetrazine derivatives were obtained and fully characterized. These derivatives were equipped with both substituents for solubility and functional groups for anchoring the tetrazine unit to the polymer backbone. A convenient method for grafting the tetrazine moiety onto polymers with pendant carboxylic groups was also developed.

The first examples of water-soluble polymers with various architectures, including linear and cross-linked microgels, modified with tetrazine units, were obtained and characterized using UV–Vis spectroscopy, voltammetry, and dynamic light scattering techniques.

The redox activity of the new low-molecular-weight tetrazines in water is associated with a one-step two-electron reduction process coupled with the transfer of two protons. These compounds demonstrated chemical reversibility in an aqueous solution containing acetate buffer, with a reduction potential that can be adjusted by substituents in the tetrazine core, ranging from −0.1 to −0.05 V vs. AgCl/Ag. We have also demonstrated that the choice of electrode material is crucial for the reaction kinetics of tetrazines. Notably, annealing the carbon electrode appeared to be the most beneficial, likely due to the formation of oxygen groups on the surface that enhance the reaction kinetics facilitating the protonation steps in the tetrazine reduction process.

Importantly, the linear polyacrylic acid and microgels with tetrazine pendant groups exhibited well-pronounced redox activity. This is a significant result, given the limited availability of water-soluble polymeric anodic materials. The cathodic process in the polymer is chemically reversible, but the electrochemical reaction is relatively slow. Therefore, the proper selection of the working electrode material is important. Thus, for the tetrazine-grafted microgels, prominent redox activity was observed on the annealed carbon paper electrode, whereas no observable electrochemical activity for the tetrazine-modified microgel on a glassy carbon electrode was detected. It is more likely attributed to the drastic impediment of the protonation step, which is essential for the electrochemical activity of the tetrazine moiety in an aqueous solution. In contrast, the oxygen groups that are present on the carbon paper surface after annealing in the air may enhance the electrode process by participating in the protonation steps involved in the tetrazine reduction reaction.

To summarize, the reported new water-soluble tetrazine derivatives, both low-molecular-weight and polymer variants, expand the scope of anodic materials available for aqueous organic redox flow batteries.

## Figures and Tables

**Table 1:** The hydrodynamic radii (R_h_) and the radii of gyration (R_g_) of PAA, PAA-TZ **10,** the MG-TZ, and MG in water at different pH and temperatures.

Code	pH	T = 23 °C			T = 50 °C		
		R_h_, nm	R_g_, nm	R_g_/R_h_	R_h_, nm	R_g_, nm	R_g_/R_h_
PAA	2.4	4 ± 1	–	–	3 ± 1	–	–
	6.5	7 ± 1	–	–	7 ± 1	–	–
	9.1	8 ± 1	–	–	7 ± 1	–	–
PAA-TZ	2.4	13 ± 2	–	–	12 ± 2	–	–
	6.5	34 ± 3	–	–	34 ± 3	–	–
	9.1	35 ± 3	–	–	36 ± 3	–	–
MG	2.4	159 ± 2	98 ± 2	0.62 ± 0.02	– *	– *	– *
	6.5	356 ± 7	140 ± 5	0.39 ± 0.02	286 ± 7	163 ± 8	0.57 ± 0.03
	9.1	388 ± 5	145 ± 7	0.37 ± 0.02	341 ± 5	140 ± 8	0.41 ± 0.02
MG-TZ	2.4	206 ± 8 *	– *	– *	– *	– *	– *
	6.5	120 ± 5	105 ± 9	0.87 ± 0.02	122 ± 6	109 ± 11	0.89 ± 0.02
	9.1	118 ± 5	107 ± 10	0.90 ± 0.02	119 ± 5	107 ± 10	0.89 ± 0.02

* Precipitation appears in equilibrium conditions.

**Table 2:** The peak potentials, peak separation, and half-wave potentials of tetrazines **3**, **6**, **7**, and **9** vs. Ag/AgCl/KCl_sat._ in a 0.2 M acetate buffer solution pH 4.4 at a Pt electrode at a scan rate of 25 mV/s.

No.	Structure	E_p,c_, V	E_p,a_, V	(E_p,a_ − E_p,c_), V	(E_p,c_ + E_p,a_)/2, V	|I_p,c_/I_p,a_|
**3**	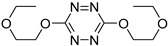	–0.131	0.034	0.165	–0.049	0.7
**7**	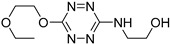	–0.270	0.103	0.373	–0.084	0.8
**6**	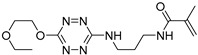	–0.261	0.066	0.327	–0.098	0.9
**9**	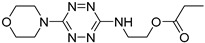	–0.222	0.062	0.284	–0.080	0.9

## Data Availability

The original contributions presented in this study are included in the article/Supplementary Materials. Further inquiries can be directed to the corresponding authors.

## Supplementary Materials

The following supporting information can be downloaded at: https://www.mdpi.com/article/10.3390/polym17010060/s1, Figures S1–S12: NMR spectra; Figures S13–S18: HRMS spectra; Figures S19 and S20: UV-Vis spectra; Figures S21 and S22: DLS data; Figure S23: SEC curve; Figure S24: CV of tetrazine.
