# Supplementary material for: Redox-Active Water-Soluble Low-Weight and Polymer-Based Anolytes Containing Tetrazine Groups: Synthesis and Electrochemical Characterization
Source: Polymers (Basel). 2024 Dec 29;17(1):60. doi: 10.3390/polym17010060 (PMC11722628; doi:10.3390/polym17010060)
Supplement: Supplementary file 1 [file polymers-17-00060-s001.zip › polymers-3396721-supplementary.pdf]

Supporting Information

Redox-Active, Water-Soluble Low-Weight Polymer-Based Anolytes Containing Tetrazine Groups: Synthesis and Electrochemical Characterization

Elena Yu. Kozhunova [1,2,*], Vyacheslav V. Sentyurin [1,2], Alina I. Inozemtseva [1,3], Anatoly D. Nikolenko [1,3], Alexei R. Khokhlov [1] and Tatiana V. Magdesieva [2,*]

[1] Physics Department, Lomonosov Moscow State University, Moscow 119991, Russia; sentyurinvv@gmail.com (V.V.S.); a.i.inozemtseva@yandex.ru (A.I.I.); nikolenko2001@gmail.com (A.D.N.); khokhlov@polly.phys.msu.ru (A.R.K.)
[2] Chemistry Department, Lomonosov Moscow State University, Moscow 119991, Russia
[3] N.N. Semenov Federal Research Center for Chemical Physics, Moscow 119991, Russia
* Correspondence: kozhunova@polly.phys.msu.ru (E.Y.K.), tvm@org.chem.msu.ru (T.V.M.)

Contents

1. NMR spectra 2
2. HRMS spectra 8
3. Electronic spectrum of 7 11
4. Tetrazine decomposition by KPS 12
5. Size distribution of polymers by dynamic light scattering 12
6. Hydrodynamic radius of MG-TZ in different conditions 13
7. Synthesis of linear PAA 13
8. Cyclic voltammetry of tetrazine 7 on glassy carbon electrode. 14

1. NMR spectra

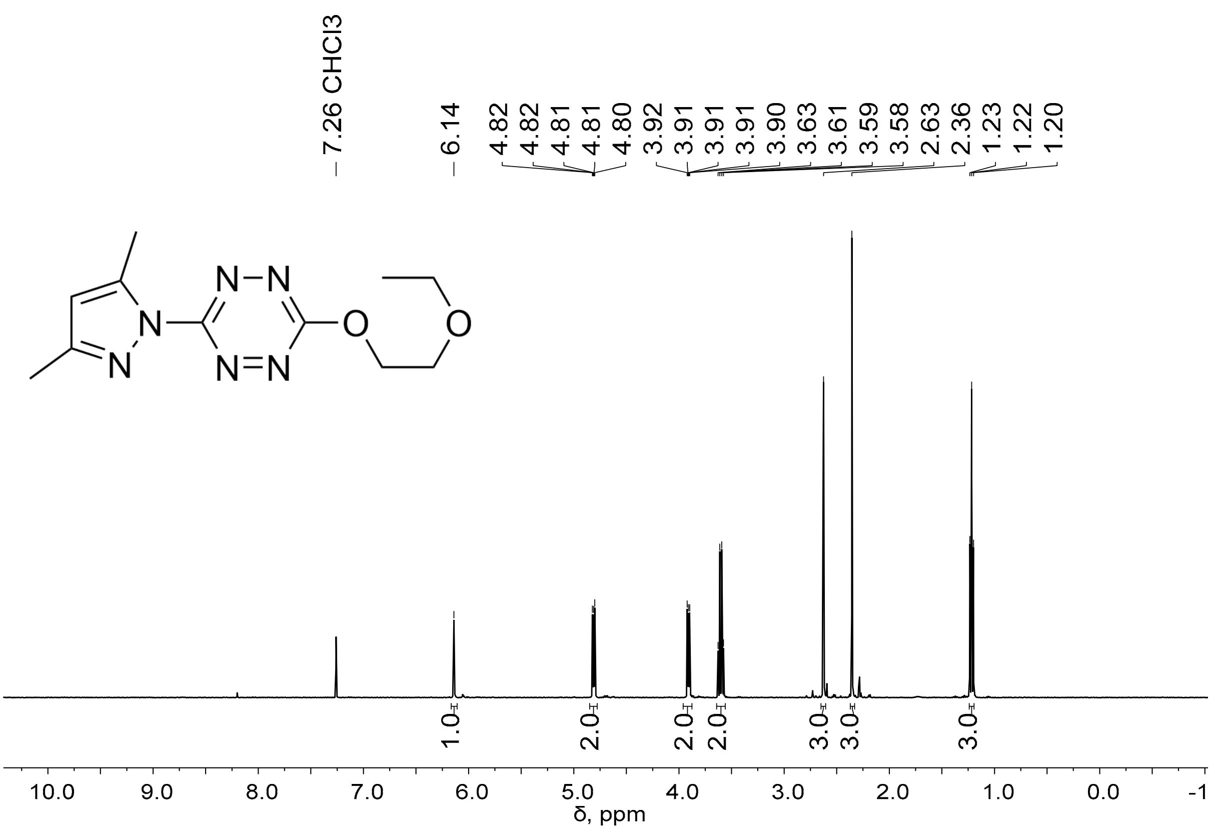

Figure S1. *^{1}H NMR spectrum of* 3-(3,5-dimethyl-1H-pyrazol-1-yl)-6-(2-ethoxyethoxy)-1,2,4,5-tetrazine *(2) in $CDCl_3$.*

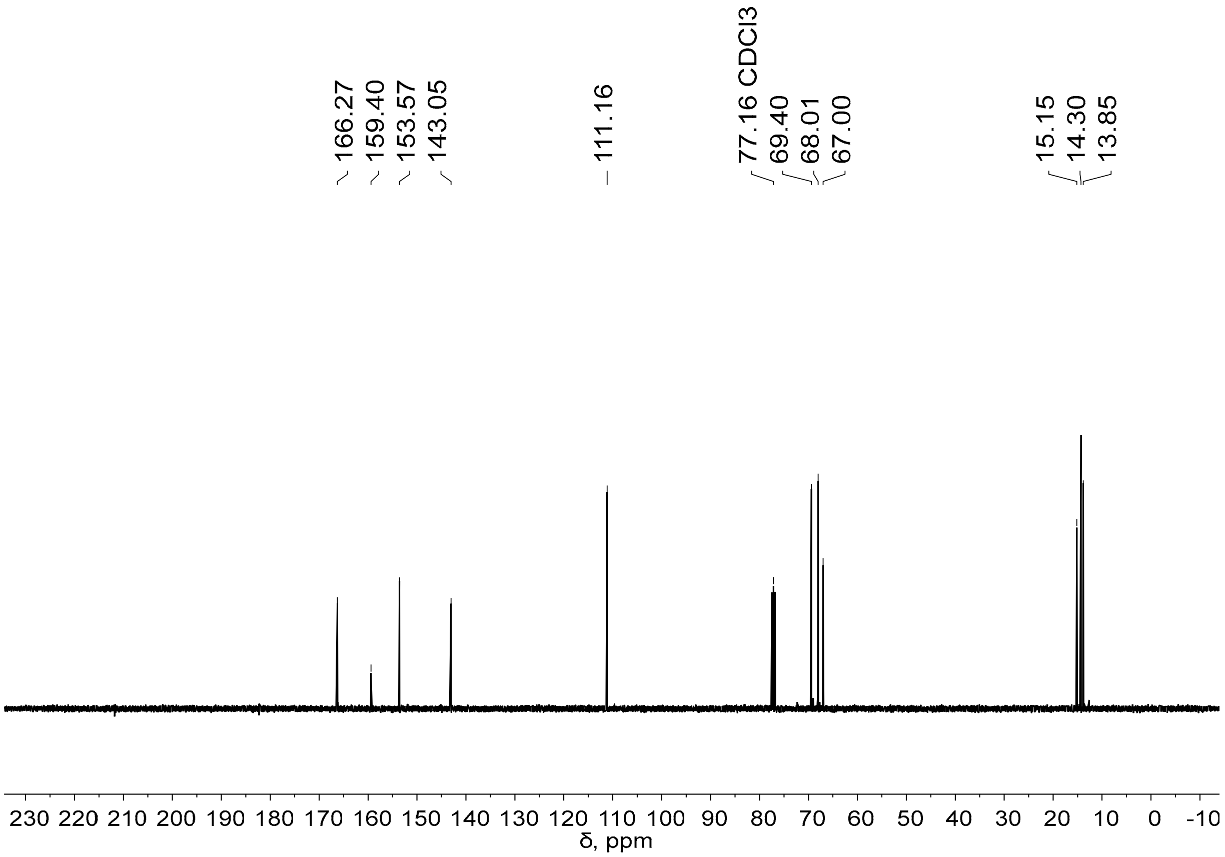

Figure S2. [13]*C NMR spectrum of* 3-(3,5-dimethyl-1H-pyrazol-1-yl)-6-(2-ethoxyethoxy)-1,2,4,5-tetrazine *(2) in* $CDCl_3$.

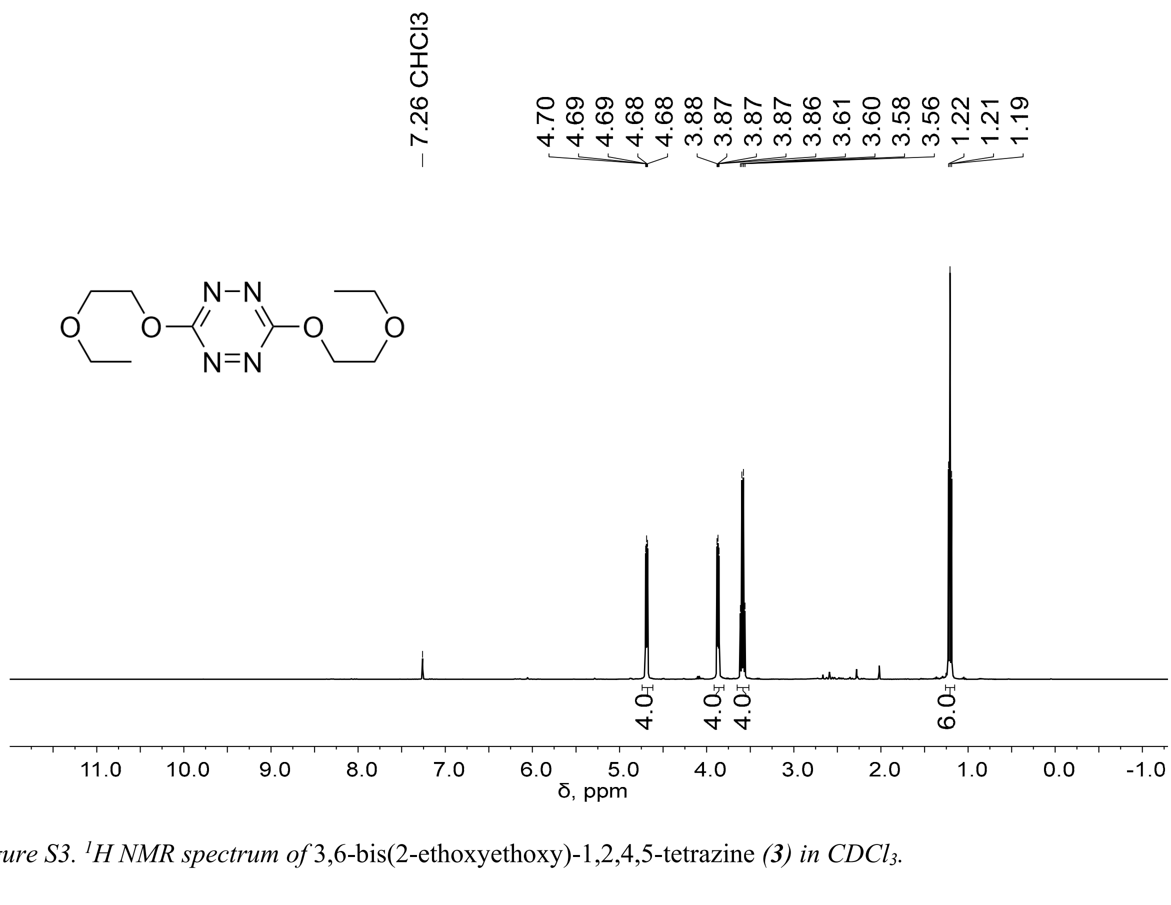

Figure S3. [1]*H NMR spectrum of* 3,6-bis(2-ethoxyethoxy)-1,2,4,5-tetrazine *(3) in* $CDCl_3$.

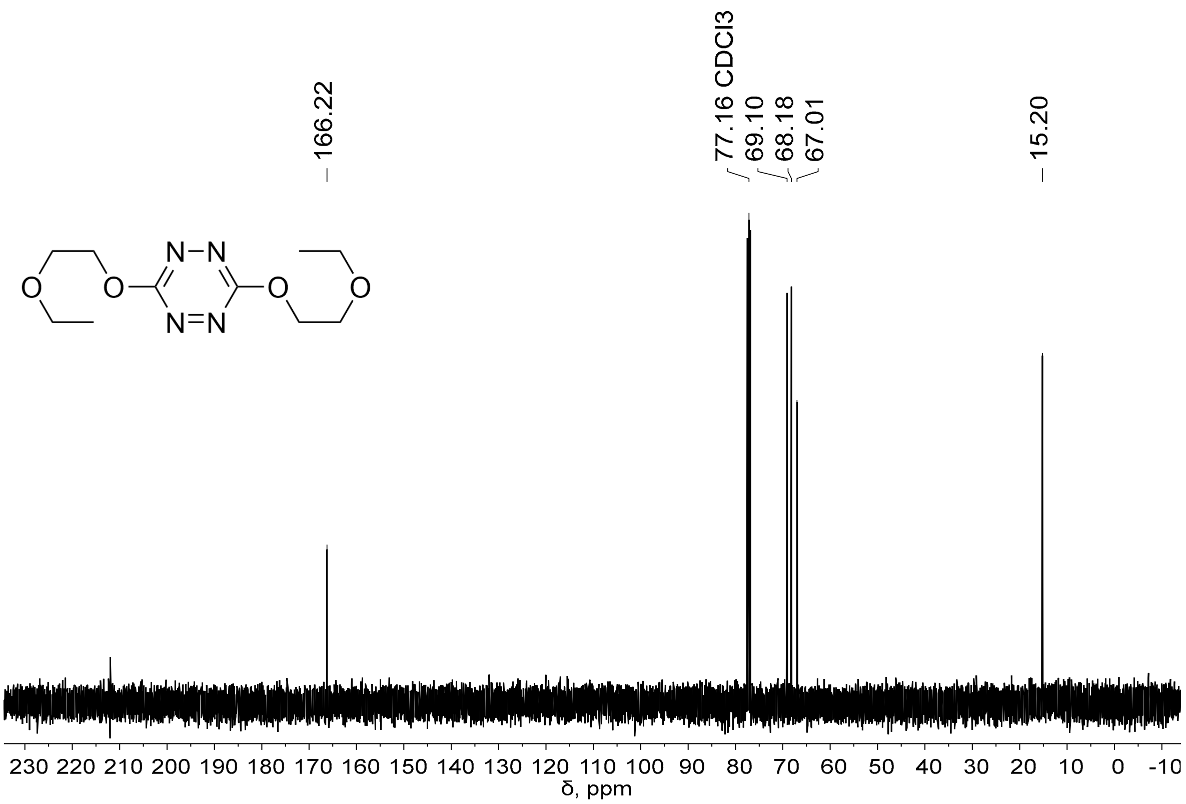

Figure S4. ^{13}C NMR spectrum of 3,6-bis(2-ethoxyethoxy)-1,2,4,5-tetrazine *(**3**) in* $CDCl_3$.

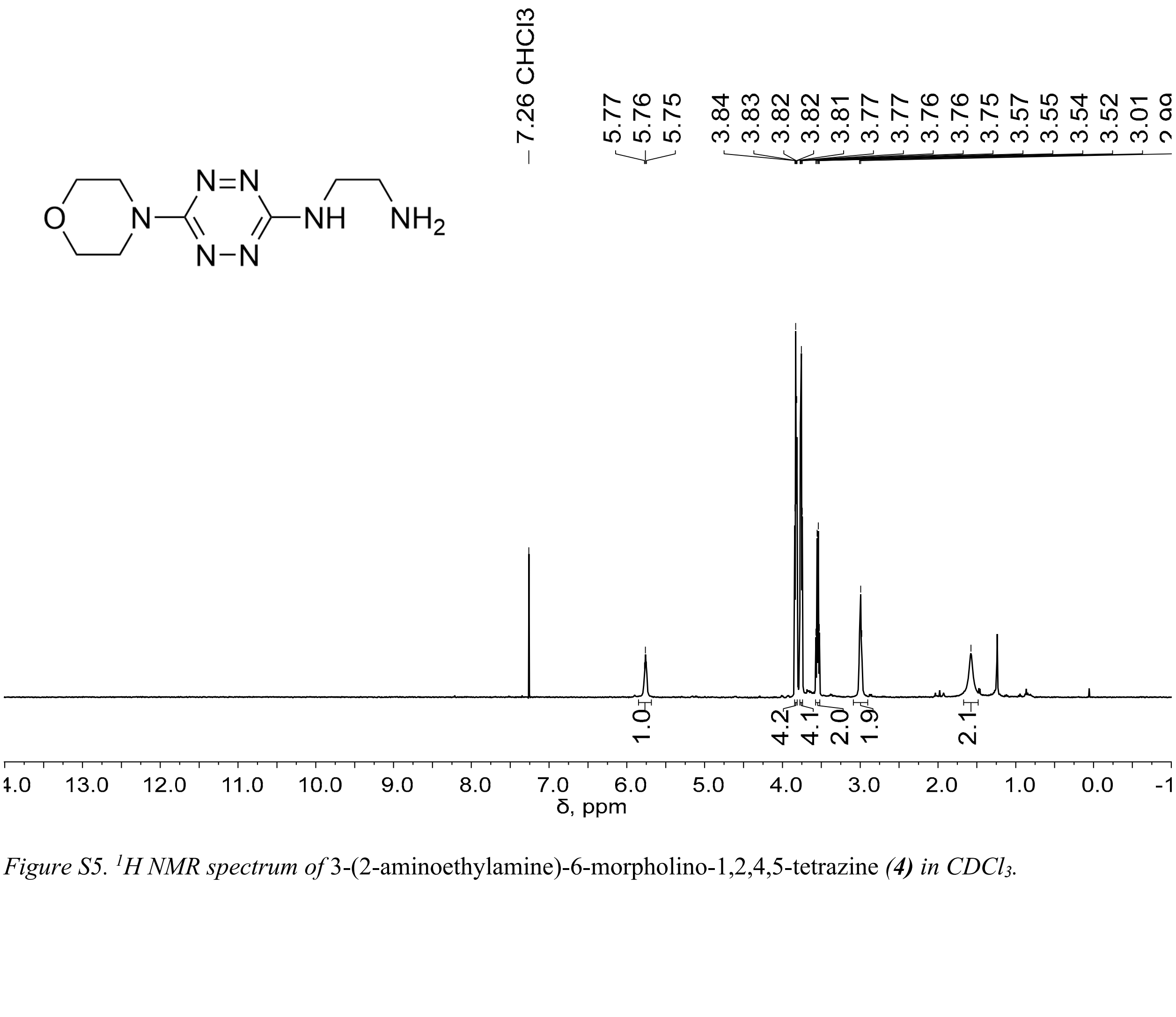

Figure S5. ^{1}H NMR spectrum of 3-(2-aminoethylamine)-6-morpholino-1,2,4,5-tetrazine *(**4**) in* $CDCl_3$.

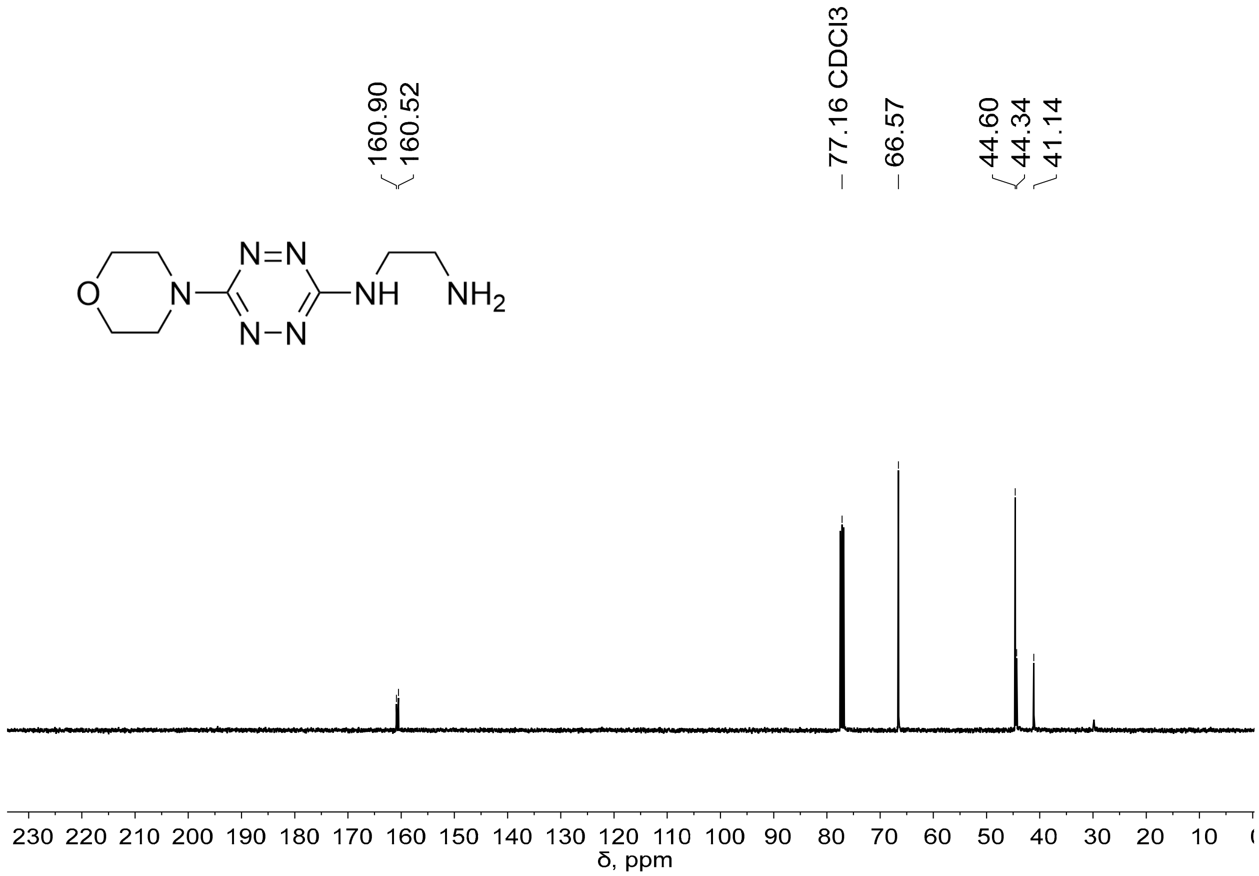

Figure S6. ^{13}C NMR spectrum of 3-(2-aminoethylamine)-6-morpholino-1,2,4,5-tetrazine *(**4**) in $CDCl_3$.*

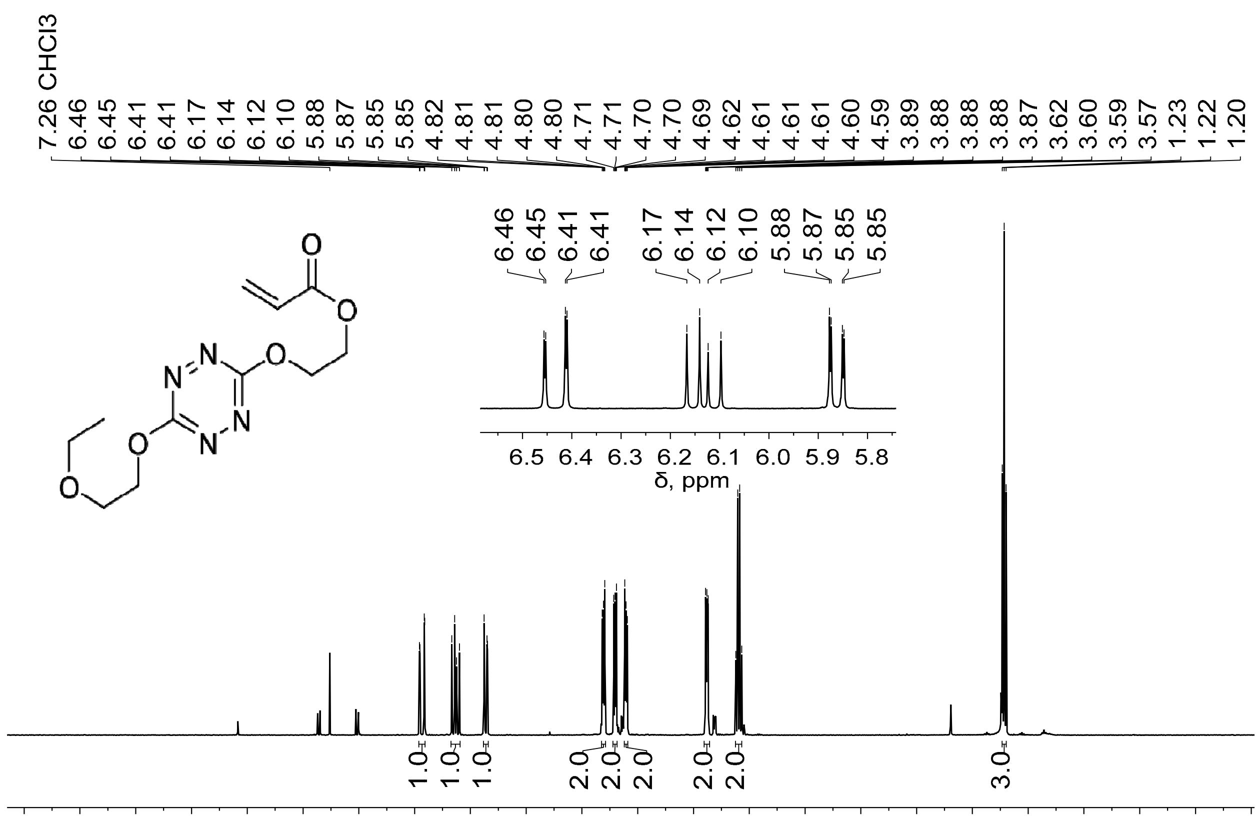

*Figure S7. ^{1}H NMR spectrum of **5** in $CDCl_3$.*

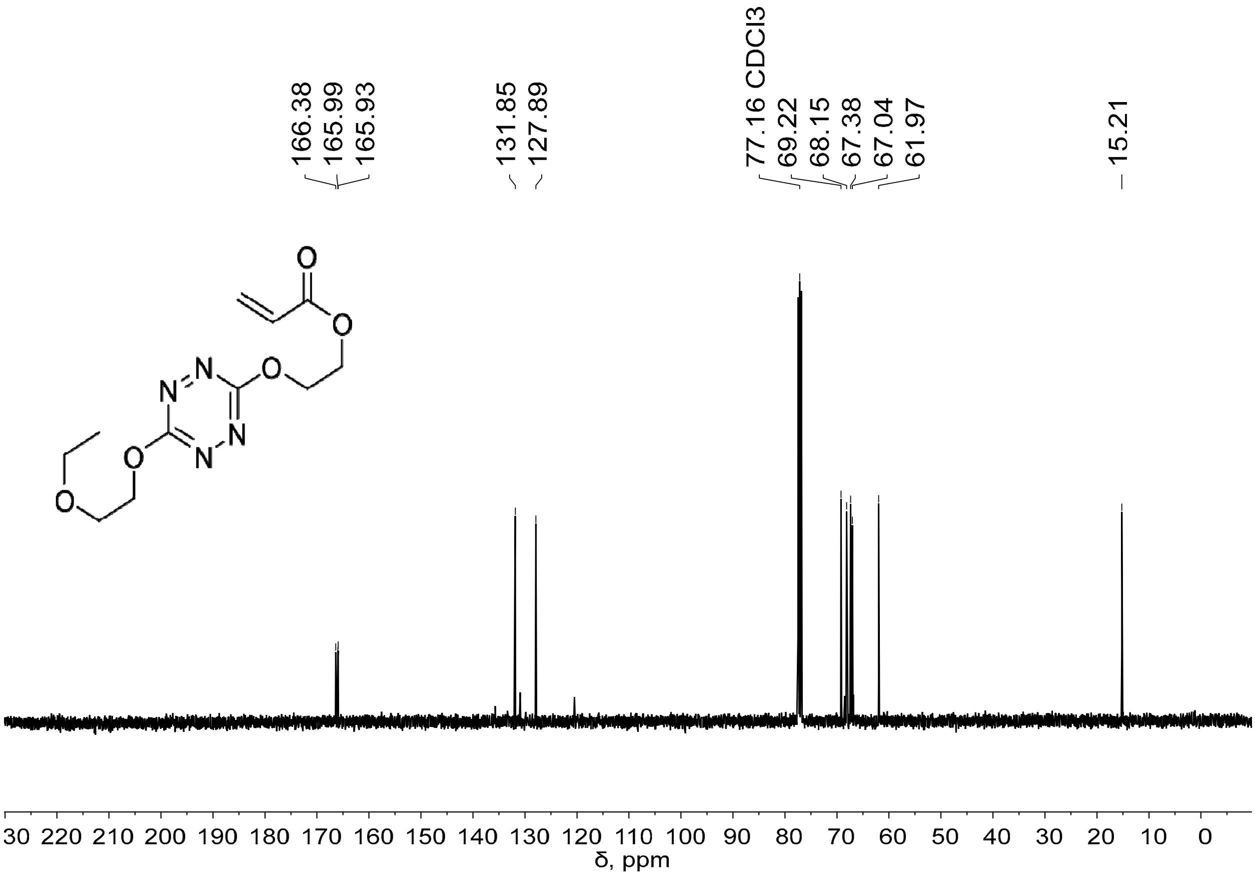

Figure S8. ^{13}C *NMR spectrum of* ***5*** *(LS-329) in* $CDCl_3$.

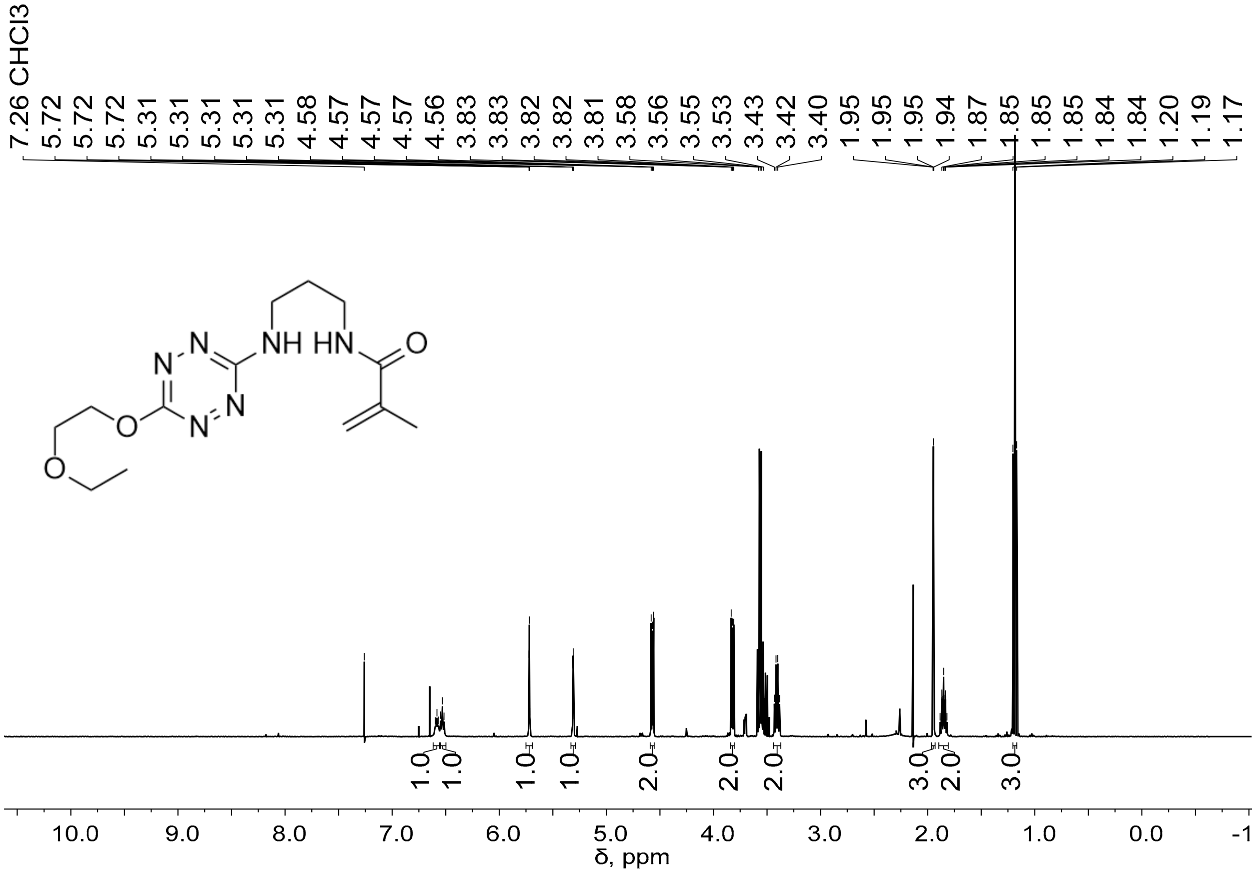

Figure S9. ^{1}H *NMR spectrum of N-(3-((6-(2-ethoxyethoxy)-1,2,4,5-tetrazin-3-yl)amino)propyl)methacrylamide* (***6***) *in* $CDCl_3$.

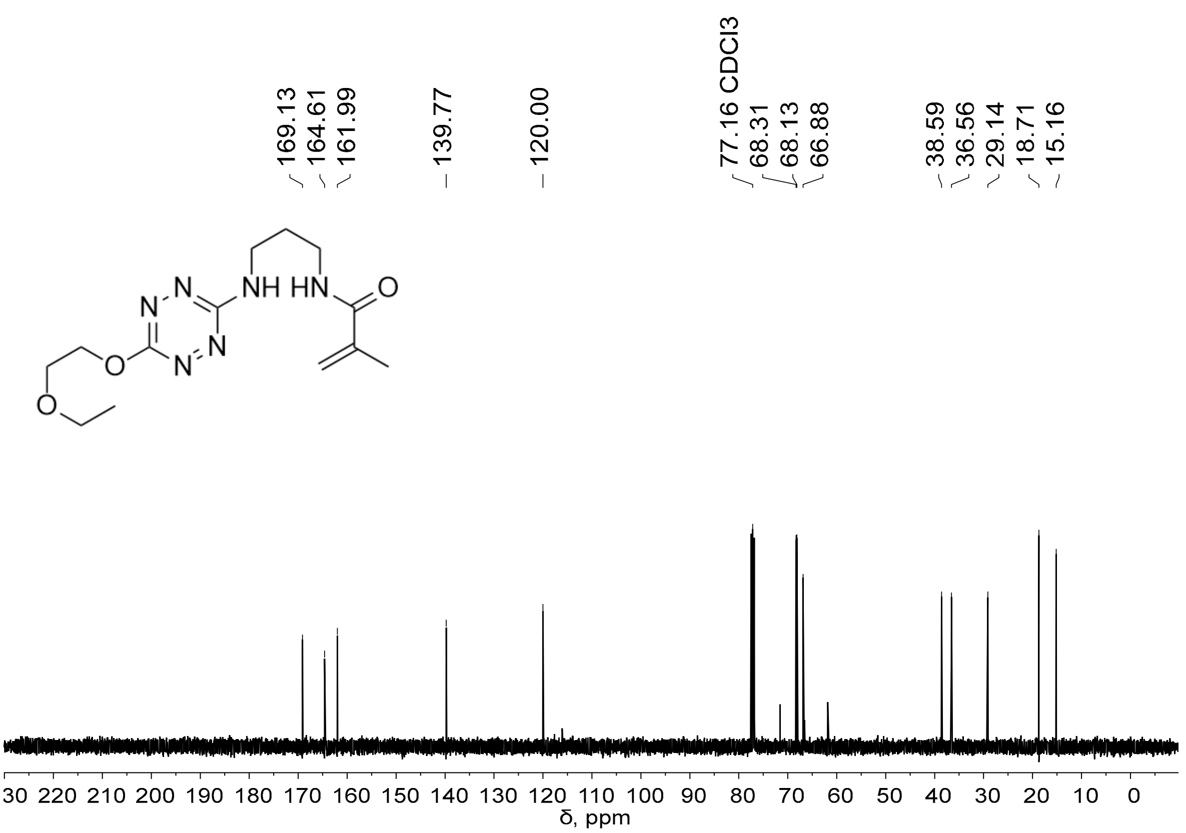

*Figure S10. ^{13}C NMR spectrum of N-(3-((6-(2-ethoxyethoxy)-1,2,4,5-tetrazin-3-yl)amino)propyl)methacrylamide (**6**) in CDCl$_3$.*

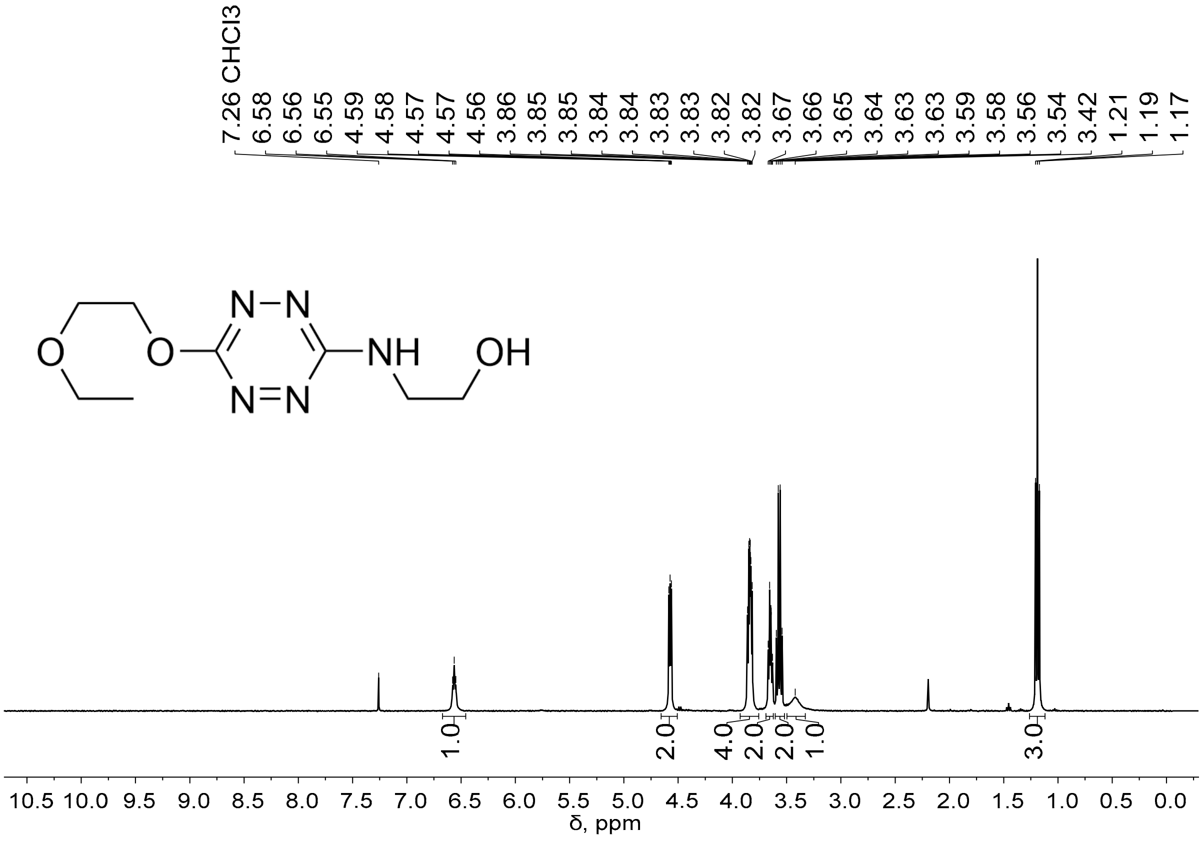

Figure S11. ^{1}H NMR spectrum of 2-((6-(2-ethoxyethoxy)-1,2,4,5-tetrazin-3-yl)amino)ethan-1-ol (**7**) *in CDCl$_3$.*

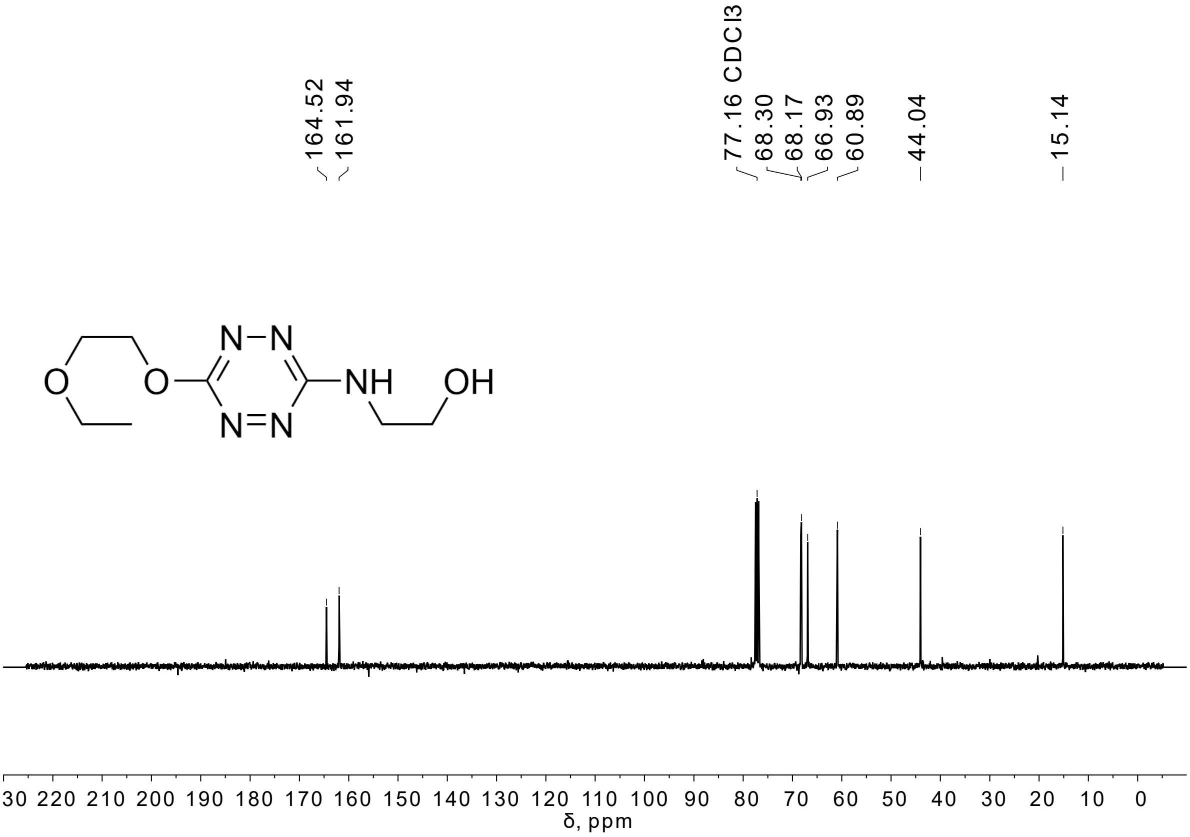

Figure S12. ^{13}*C NMR spectrum of* 2-((6-(2-ethoxyethoxy)-1,2,4,5-tetrazin-3-yl)amino)ethan-1-ol (**7**) *in* $CDCl_3$.

2. HRMS spectra

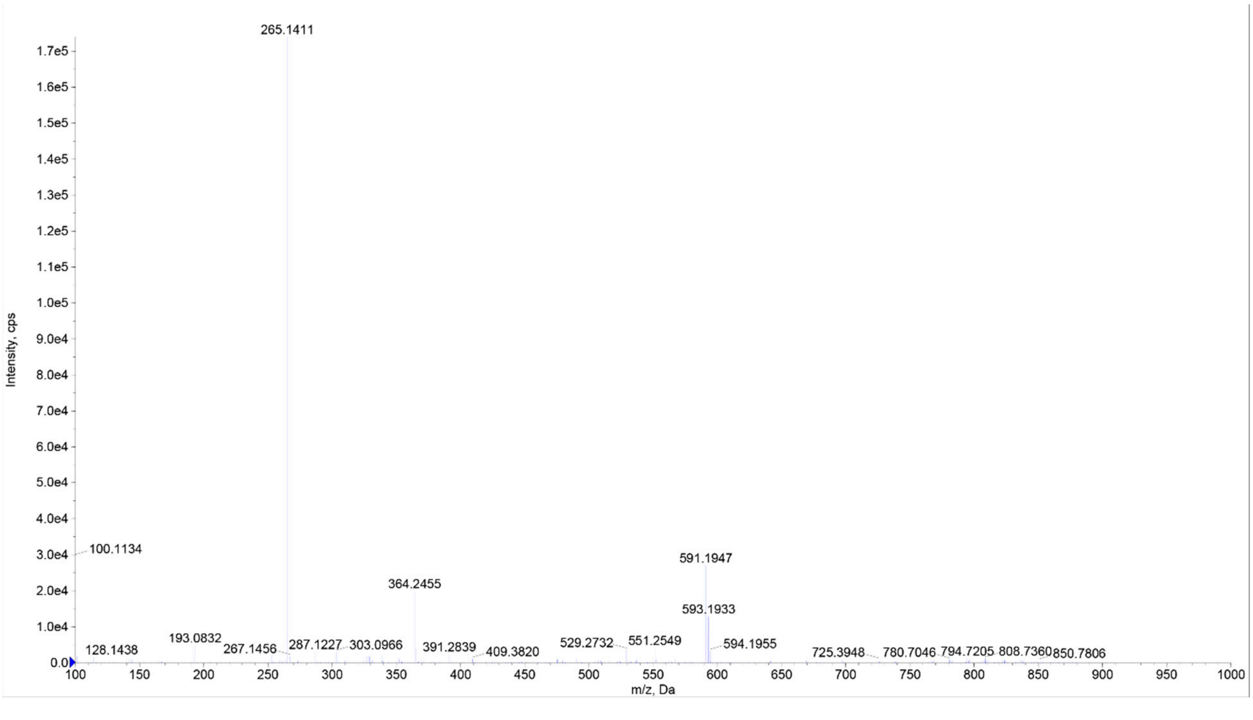

Figure S13. HRMS spectrum of ***2***.

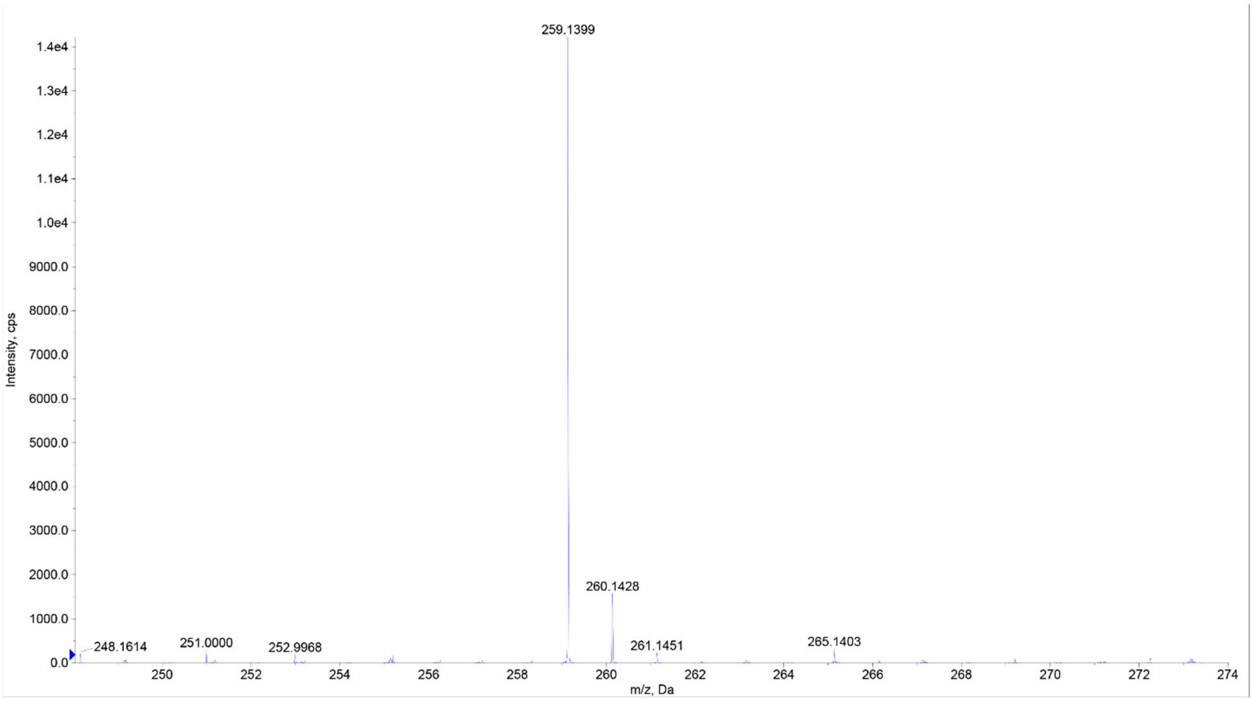

*Figure S14. HRMS spectrum of **3**.*

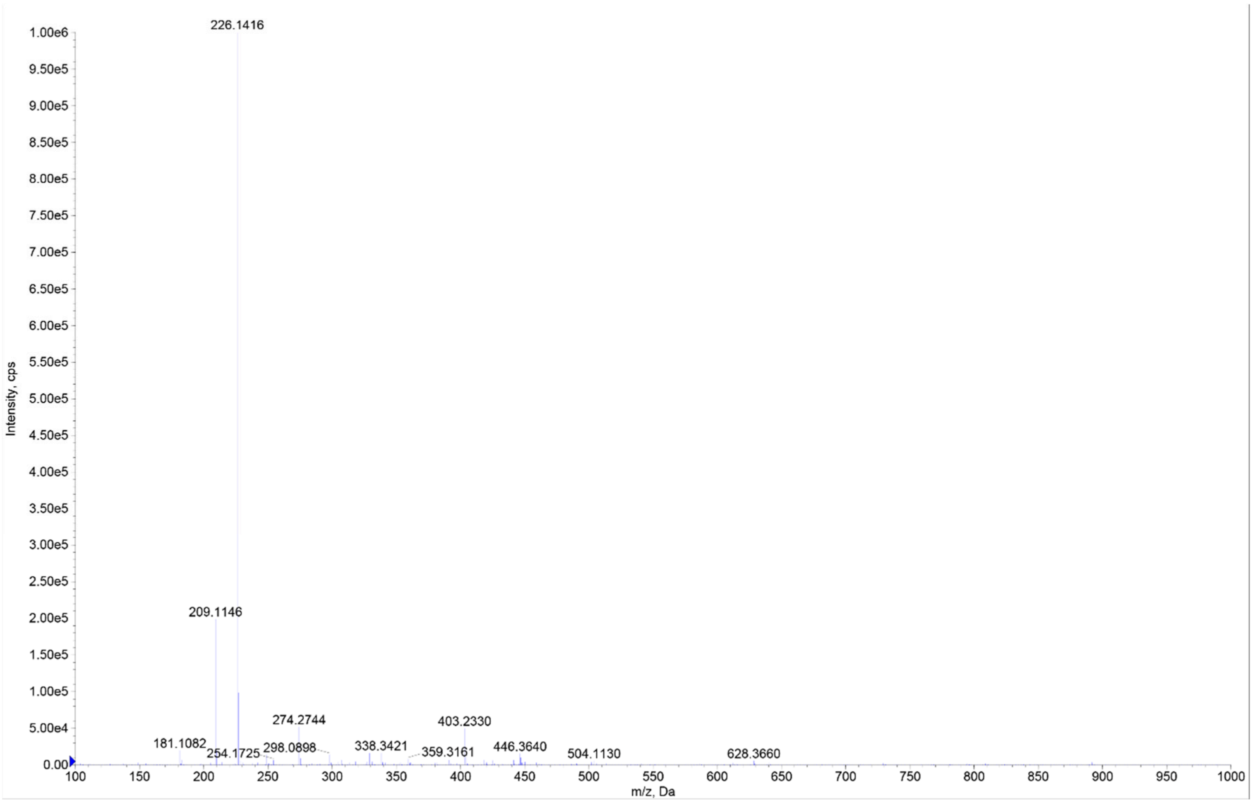

*Figure S15. HRMS spectrum of **4**.*

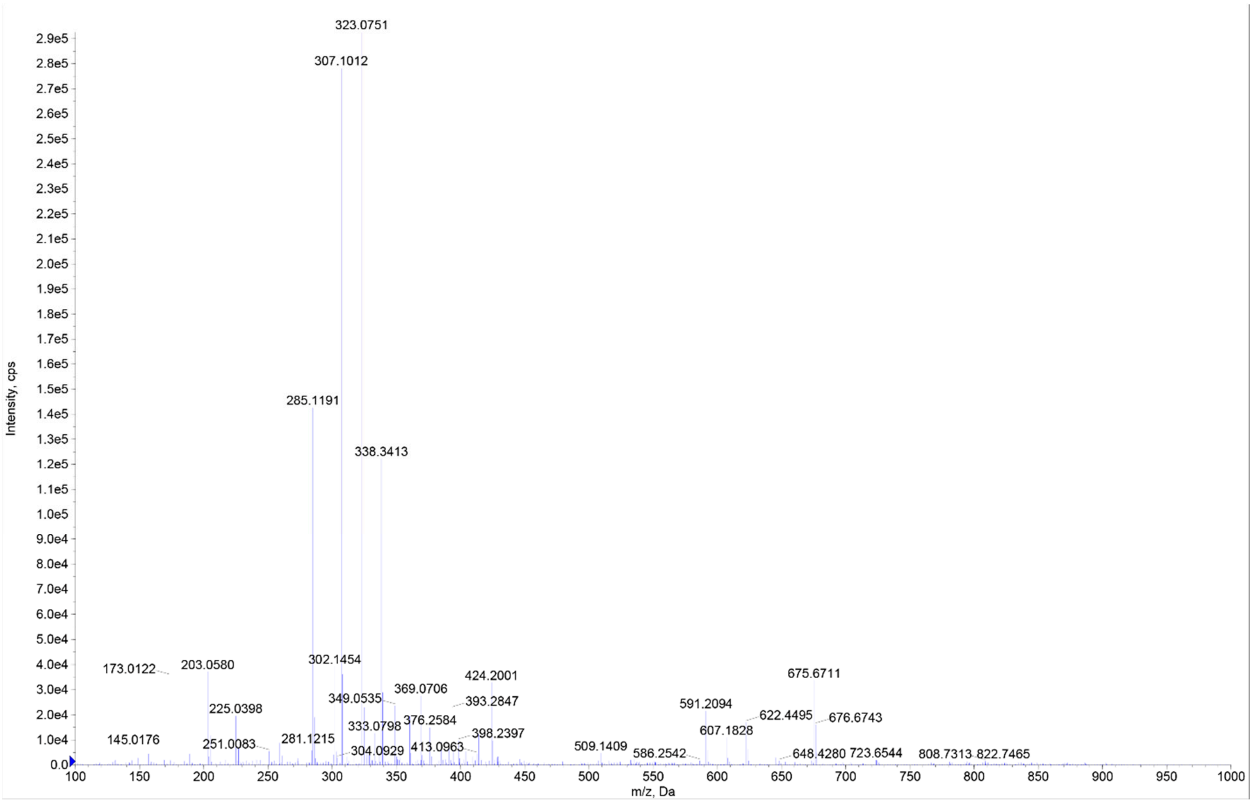

*Figure S16. HRMS spectrum of **5**.*

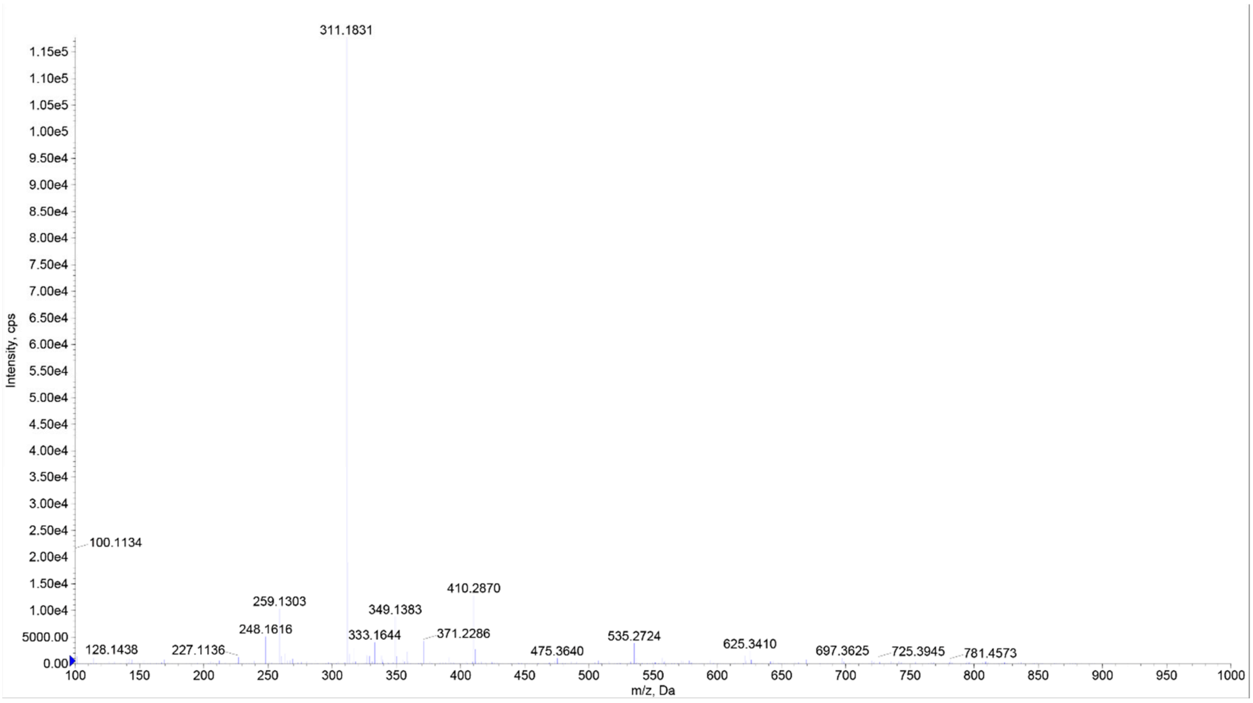

*Figure S17. HRMS spectrum of **6**.*

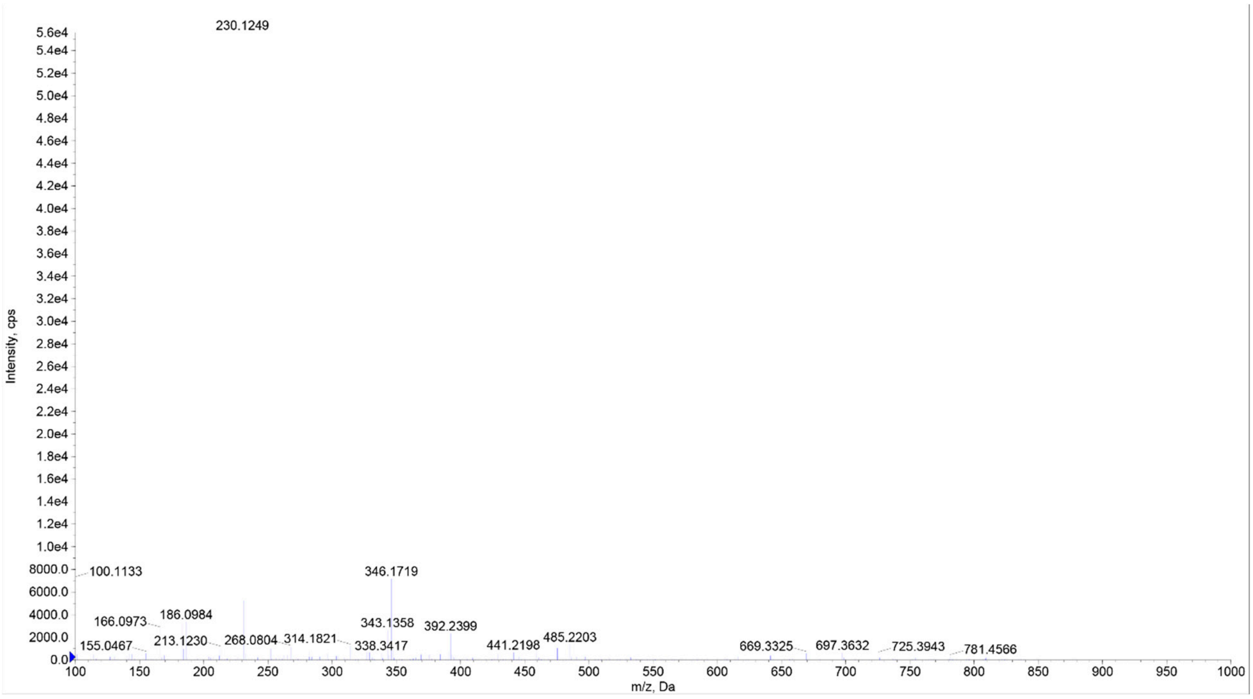

Figure S18. HRMS spectrum of 7.

3. UV-Vis of 7

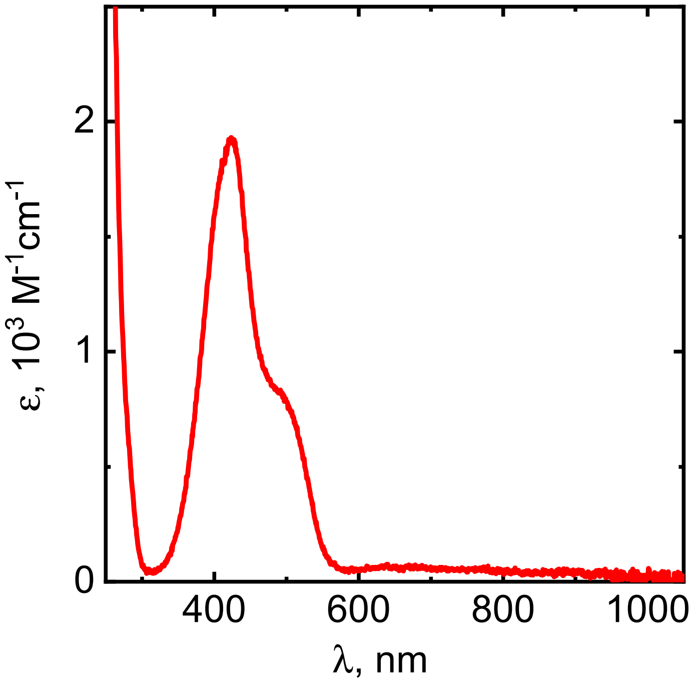

Figure S19. UV-Vis spectrum of 7.

4. Tetrazine decomposition by KPS

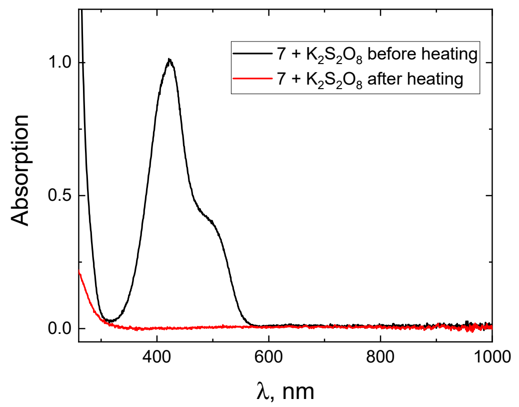

*Figure S20. The UV-vis spectra of solution of tetrazine **7** (0.9 mM) and $K_2S_2O_8$ (9.6 mM) in water before (black line) and after heating at 80 °C for 0.5 hour (red line).*

5. Size distribution of polymers by dynamic light scattering

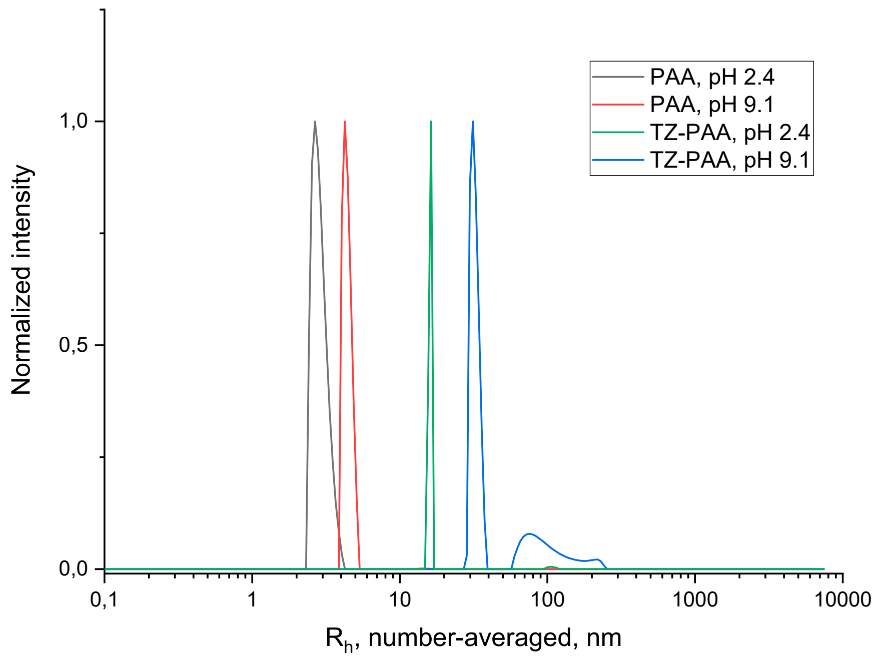

Figure S21. Hydrodynamic radii R_h distribution for linear polyacrylic acid before (PAA) and after (TZ-PAA) modification with tetrazine groups. The measurements were performed at the scattering angle of 90 °, at temperature T = 21 °C in 0.1 M KCl aqueous solution.

6. Hydrodynamic radius of MG-TZ in different conditions

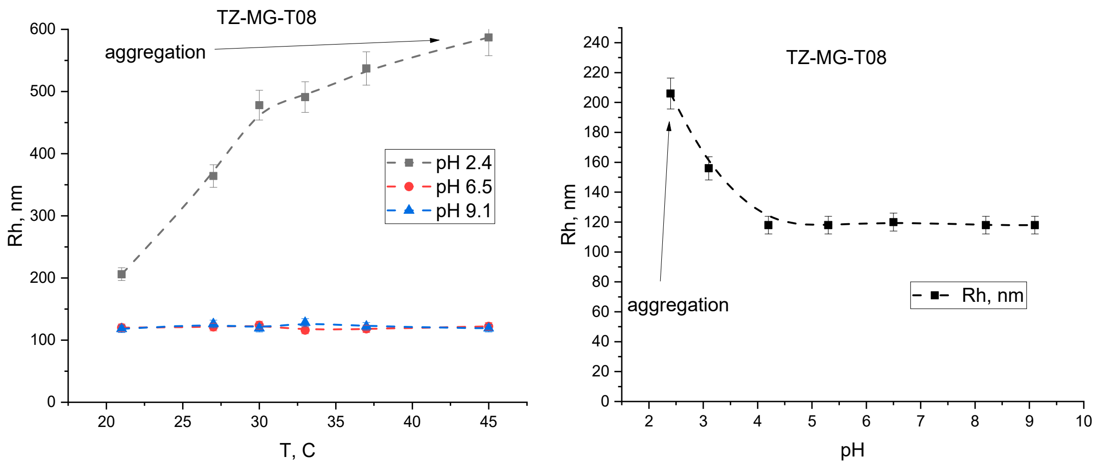

Figure S22. The dependence of MG-TZ R_h on temperature (left) and pH (right) of aqueous solution.

7. Synthesis of linear PAA

The synthesis of polyacrylic acid using the RAFT-mechanism proceeded by the following routine. The reaction mixture for polymerization was prepared by dissolving the required amount of the initiator AIBN (10^{-3} M) and the RAFT agent (benzyl dithiobenzoate) (0.1 M) in the freshly distilled acrylic acid (AA) monomer mixture containing DMF. The molar ratio of AA and DMF was equal to 1 : 3. Solution was poured into the ampoule, degassed by three freeze-pump-thaw cycles, and sealed. The ampoule was immersed into a water bath which was preheated at 80 °C for 48 hours. Then, the sample was cooled in liquid nitrogen; the polymer was dissolved in a ten-fold excess of 1,4-dioxane and dried several times by lyophilization under a vacuum.

For size exclusion chromatography (SEC), the polymer was modified by methylation of the carboxylic acid groups using diazomethane. The molecular weight characteristics of the polymers were studied by SEC (Figure S21). The SEC measurements were performed in THF at 40 °C with a flow rate of 1.0 mL min^{-1} using a Shimadzu liquid chromatograph equipped with a refractive index and UV-detectors and two columns packed with styragel with pore dimensions of 10^4 and 10^5 Å. The SEC system was calibrated using narrow dispersed linear polystyrene standards. Calculations were carried out using “LCsolution” software.

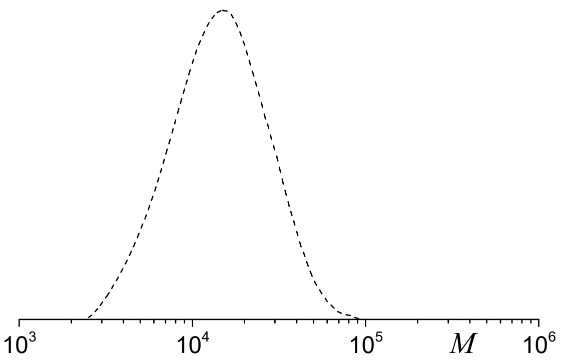

Figure S23. The SEC curve normalized to the unit area for the linear PAA.

8. Cyclic voltammetry of tetrazine 7 on glassy carbon electrode.

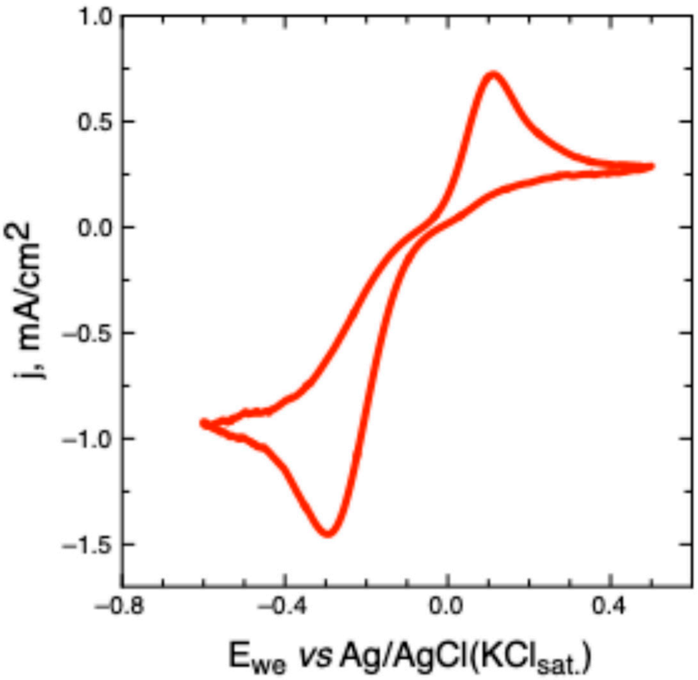

Figure S24. Cyclic voltammogram of 12 mM solution of tetrazine 7 in 0.2 M acetate buffer (scan rate 25 mV/s) on glassy carbon electrode.
